# Transcriptome screening followed by integrated physicochemical and structural analyses for investigating RNA-mediated berberine activity

**DOI:** 10.1093/nar/gkab189

**Published:** 2021-03-30

**Authors:** Sagar Satpathi, Tamaki Endoh, Peter Podbevšek, Janez Plavec, Naoki Sugimoto

**Affiliations:** Frontier Institute for Biomolecular Engineering Research (FIBER), Konan University, 7-1-20 Minatojima-minamimachi, Kobe 650-0047, Japan; Frontier Institute for Biomolecular Engineering Research (FIBER), Konan University, 7-1-20 Minatojima-minamimachi, Kobe 650-0047, Japan; Slovenian NMR Centre, National Institute of Chemistry, Hajdrihova 19, Ljubljana SI-1000, Slovenia; Slovenian NMR Centre, National Institute of Chemistry, Hajdrihova 19, Ljubljana SI-1000, Slovenia; EN→FIST Centre of Excellence, Trg OF 13, SI-1000 Ljubljana, Slovenia; Faculty of Chemistry and Chemical Technology, University of Ljubljana, Večna pot 113, p. p. 537, SI-1000 Ljubljana, Slovenia; Frontier Institute for Biomolecular Engineering Research (FIBER), Konan University, 7-1-20 Minatojima-minamimachi, Kobe 650-0047, Japan; Graduate School of Frontiers of Innovative Research in Science and Technology (FIRST), Konan University, 7-1-20 Minatojima-minamimachi, Kobe 650-0047, Japan

## Abstract

Non-coding RNAs are regarded as promising targets for the discovery of innovative drugs due to their abundance in the genome and their involvement in many biological processes. Phytochemicals (PCs) are the primary source of ligand-based drugs due to their broad spectrum of biological activities. Since many PCs are heterocyclic and have chemical groups potentially involved in the interaction with nucleic acids, detailed interaction analysis between PCs and RNA is crucial to explore the effect of PCs on RNA functions. In this study, an integrated approach for investigating interactions between PCs and RNAs were demonstrated to verify the RNA-mediated PCs functions by using berberine (BRB) as a model PC. RNA screening of a transcriptome library followed by sequence refinement found minimal RNA motif consisting of a cytosine bulge with U-A and G-U neighbouring base pairs for interaction with BRB. NMR-based structure determination and physicochemical analyses using chemical analogues of BRB demonstrated the importance of electrostatic and stacking interactions for sequence selective interaction and RNA stabilization. The selective interaction with a relatively small RNA motif based on a chemical structure of a planer heterocyclic highlights the biological activities of various PCs mediated by the interactions with particular functional RNAs. In addition, the systematic and quantitative investigations demonstrated in this study could be useful for the development of therapeutic chemicals targeting functional RNAs, based on the PCs, in the future.

## INTRODUCTION

Currently, therapeutic drugs exist mainly in the form of target proteins (1). However, due to the limited number of druggable proteins involved in diseases, the discovery of new drug targets in proteins is decreasing (2–4). Nearly 700 proteins among ∼20 000 expressed human proteins have been targeted by drugs (5,6). Recent genomics studies, such as ENCODE (7) and FANTOM (8) projects, have suggested that the majority of transcribed RNAs in the human genome does not result in protein expression. Of the 75% region of the human genome, which is transcribed into RNA, only 1–2% of the RNAs encode proteins (5,9). Simultaneously, non-coding RNA transcripts and non-coding regions on coding mRNAs that have been previously considered meaningless are increasingly thought to play important roles in biological activities (10). Therefore, the development of therapeutic drugs targeting RNAs, including both coding and non-coding regions, shows the potential to broaden the scope of drug discovery (5).

RNAs with biologically important functions have been studied extensively from a structural viewpoint. RNAs form unique and complex secondary and tertiary structures, which are indispensable for their function (11,12). Although the chemical units of RNAs, which consist of four kinds of ribonucleotides, have less variety compared to those of proteins, the unique organization of the ribonucleotides in the tertiary structures can be a recognition site for small chemical compounds (5). Therefore, in recent years, studies of chemical library screening and rational molecular design targeting specific RNA structures have been actively pursued. For example, Disney *et al.* identified the preferred RNA structure for a particular ligand from 3 000 000 combinations of RNA motif–ligand interactions (13). Furthermore, the Inforna platform, a large database for storing interactions between small molecules and RNA based on the screening results, is making it possible to select interacting chemicals for objective RNA sequences (14). On the other hand, natural systems of gene modulation exist through interactions between RNA and metabolite chemicals (15), although they are not considered medicinal chemicals. A specific region on mRNA, the so-called riboswitch, modulates downstream gene expression by stabilizing the RNA tertiary structure upon the interaction of a specific metabolite in cells (16). Since the modulation of gene expression by riboswitches is fundamental for prokaryotic activity, the metabolite binding region of riboswitches could be an attractive target for antibiotic development (17). These findings indicate that natural chemical compounds may potentially modulate RNA functions through direct interaction with RNAs.

Phytochemicals (PCs), which are often derived from the secondary metabolites of plants, show a broad spectrum of biological activities, such as antioxidant, antibacterial and antitumor properties (18,19). PCs are considered to affect protein functions via their chemical groups, which form various types of interactions with amino acid residues in proteins (20). Thus, PCs have been utilized as seeding molecules and have led to many successes in the development of therapeutic drugs (21,22). However, the biological importance of PCs is still not evident, especially in the context of their influence on nucleic acids. Given the active roles of nucleic acid structures in gene expression processes, such as replication, transcription, translation and post-translational protein folding (12,23–25), and those of small chemicals in the modulation of functional nucleic acids, such as riboswitches (17), some of the biological activities of PCs are likely to result from their interaction with nucleic acids. In addition, the chemical structural properties of PCs, which contain multiple heterocycles with nitrogen atoms, correspond to those of chemicals that tend to interact with RNAs rather than proteins (26). There are many reports highlighting the interaction of PCs with RNAs (27). For example, berberine (BRB), which is an alkaloid that has potent antibacterial and antitumor activities (28), is known to interact with several structured nucleic acids, such as duplex (29,30), triplex (31), transfer RNA (tRNA) (32) and G-quadruplex (33). However, few studies have systematically and quantitatively investigated the selectivity of the sequence or structural features of RNA. A greater understanding of the chemical properties of PCs interacting with RNA could reveal the mechanism of biological activity of PCs, providing useful information for the development of therapeutic drugs that directly target RNAs. The main challenge involves identifying the sequences or structural units of RNAs with high specificity and affinity to a particular PC.

Regarding the screening of RNAs that interact with small chemicals, we recently developed a technique for constructing RNA-capturing microsphere particles (R-CAMPs) (34,35). R-CAMPs consist of particles with micrometer size that display identical sequences of DNA and RNA on their surface. Each particle displays different sequence derived from the arbitrary designed library, including randomized sequences. In the previous study, we have displayed light-up aptamer libraries, which were partially randomized, and selected functional RNAs that emit fluorescence of small chemical through sorting of R-CAMPs by fluorescence-activated cell sorting (FACS) (34,35). Since many PCs have multiple heterocyclic structures and exhibit intrinsic fluorescent properties (36), we hypothesized that RNAs interacting with such PCs could be selected using R-CAMPs. In this study, we selected RNAs that interact with BRB, which is known to emit fluorescence in the state of a complex with nucleic acids, and demonstrated that RNAs containing single cytosine bulge (C-bulge) could selectively bind BRB with equilibrium association constants over 10^6^ M^−1^ at 25°C. Subsequently, studies of BRB with different variants of RNA secondary structures showed that neighbouring base pairs of the C-bulge, which contains one wobble base pair, are essential for the interaction. An NMR study clarified that BRB induced a well-organized duplex by stacking between the base pairs at the C-bulge site. Further investigations using BRB derivatives demonstrated the energetic correlation between the thermodynamic stability of the RNA and the ligand binding affinity, highlighting the importance of electrostatic and stacking interactions for the stabilization of RNA. The systematic and quantitative approach used in the present study to determine the RNA structure motifs for a specific PC interaction explores a mode of potential biological activity of PCs other than that targets proteins and provides beneficial information for future RNA-targeting drug discovery based on PCs.

## MATERIALS AND METHODS

### Oligonucleotides and chemical reagents

All DNA oligonucleotides used in this study were purchased from Fasmac Co., Ltd. (Kanagawa, Japan) or Japan Bio Services Co., Ltd. (Saitama, Japan). RNA oligonucleotides were purchased from Fasmac Co., Ltd. or GeneDesign, Inc. (Osaka, Japan) as HPLC purification grade. BRB was purchased from Sigma-Aldrich Co. (St. Louis, MO, USA). Coptisine (COP), palmatine (PAM) and epiberberine (EBRB) were purchased from Nagara Science Co., Ltd. (Gifu, Japan). For other chemical reagents, a grade appropriate for biochemical experiments was used.

### Preparation of R-CAMPs displaying transcriptome RNA library

Template cDNA library consisting of sequences derived from transcriptome RNA was prepared from total RNA of a human cervical cancer cell line (HeLa). A detailed procedure for preparing the cDNA library is described in supporting information. Preparation of R-CAMPs from the cDNA library was performed according to the previously established protocol with some modifications (34,35). Detailed experimental procedures of emulsion PCR and purification of microsphere particles (MPs), each of which immobilizes clones of single-stranded DNA templates derived from the cDNA library, are described in supporting information. Primer extension on the MPs was performed using a mixture of capture primers labelled and non-labelled with Alexa Fluor 647 (AF647) at a 1:4 molar ratio (Supplementary Table S1). After primer extension, the solution containing MPs was diluted 15-fold into a buffer containing 40 mM Tris-HCl (pH 8), 8 mM MgCl_2_, 5 mM DTT, 2 mM spermidine, 1 mM rNTPs, 0.01% Tween-20 and 1 U/μL T7 RNA polymerase (Takara Bio, Shiga, Japan). The transcription reaction and co-transcriptional capturing of nascent RNA transcripts on MPs were performed by incubating the reaction mixture at 25°C for 20 min. The transcription reaction was terminated by the addition of 800 nM competitive T7 promoter duplex (5′-GAAATTAATACGACTCACTATA-3′ and 5′-TATAGTGAGTCGTATTAATTTC-3′). After termination, the buffer solution in which R-CAMPs had been dispersed was replaced with 50 mM MES-LiOH (pH 7) containing 0.01% Tween-20 by three rounds of washing and centrifugation. The R-CAMPs were then kept on ice. Control MPs, which do not display RNA, were prepared by performing the same procedure without the addition of T7 RNA polymerase.

### Selection of R-CAMPs in the presence of BRB

R-CAMPs or control MPs were resuspended in a buffer containing 50 mM MES-LiOH (pH 7), 100 mM KCl, 0.5 mM MgCl_2_, 1 μM BRB and 100 ng/μl competitive RNA from bacteriophage MS2 (Roche, Penzberg, Germany), 0.1% DMSO and 0.01% Tween-20, and kept at room temperature for at least 60 min. The fluorescence signals of BRB on the R-CAMPs were analysed by fluorescence-activated cell sorting (FACS) using FACS Aria II (BD Biosciences, San Jose, CA, USA). Single particles were distinguished by a fluorescence signal of AF647 chemically modified on the capture primer. The AF647 signal was detected using a 633-nm excitation laser and a 660/20-nm bandpass filter. The fluorescence signals of BRB on each particle were analysed using a 488-nm excitation laser and a 530/30-nm bandpass filter. R-CAMPs showing strong fluorescence of BRB were selected and sorted one-by-one into the wells of a 96-well plate (Ina-optika, Osaka, Japan) containing 10 μl of H_2_O.

### Evaluation of sorted RNAs

DNA on sorted R-CAMPs was directly amplified in 96-well plates adding 10 μl of reaction solution for KOD-Plus-Ver.2 (Toyobo Co., Ltd., Osaka, Japan) containing A-primer and B-trP1 primer (6 pmol). After 20 cycles of PCR amplification, the samples were diluted 100-fold with H_2_O. Aliquots of the diluted samples (1 μl) were mixed with Thunderbird qPCR Mix (Toyobo Co., Ltd.) containing A-primer and B-trP1 primer (4 pmol). DNA amplification profiles were subsequently evaluated using the Mx3000P qPCR system to estimate the number of PCR cycles that showed signal saturation. Based on the number of PCR cycles showing saturation, template DNAs for the transcription of sorted RNAs were prepared by 20 or 24 cycles of PCR amplification using KOD-Plus-Ver.2. The second PCR products (1 μl) were diluted 10-fold in transcription buffer containing 40 mM Tris-HCl (pH 8), 8 mM MgCl_2_, 5 mM DTT, 2 mM spermidine, 1 mM rNTPs, 0.01% Tween-20 and 1 U/μl T7 RNA polymerase. The transcription of the sorted RNAs was performed at 37°C for 60 min. RNA samples in the transcription buffer were then diluted 10-fold in a buffer containing 50 mM MES-LiOH (pH 7), 100 mM KCl, 0.01% Tween-20 and 0.5 U TURBO DNase (Thermo Fisher Scientific), and then incubated at 37°C for 30 min. RNA samples (6.25 μl) were mixed with buffer (3.75 μl) containing 50 mM MES-LiOH (pH 7), 100 mM KCl, 2.67 μM BRB, 0.267% DMSO and 0.01% Tween-20. Although the mixed samples were crude, containing dNTPs, rNTPs, DNA and RNA polymerases, the mixed solution, containing 50 mM MES-LiOH (pH 7), 100 mM KCl, 0.5 mM MgCl_2_, 1 μM BRB, 0.1% DMSO and 0.01% Tween-20, was the same with the buffer at R-CAMPs sorting except for competitive RNA from bacteriophage MS2. The mixtures were incubated at 25°C for 60 min. The fluorescence signals of BRB in the mixtures were measured using a fluorescence microplate reader (Infinite M200 PRO; Tecan, Mannedorf, Switzerland) at 365 nm excitation and 550 nm emission.

### Sequence analysis

DNA templates prepared by PCR for the transcription of sorted RNAs were purified using the QIAquick PCR Purification Kit (Qiagen, Venlo, Netherlands). The purified DNA was mixed with A-primer, and the sequences were analysed using a contract service (Fasmac, Kanagawa, Japan). Sequence alignment was performed on the Basic Local Alignment Search Tool (BLAST) website.

### Evaluation of designed RNAs

DNA templates for transcribing designed RNAs and their truncates were prepared by primer extension or PCR amplification using the oligonucleotides listed in Supplementary Table S2. DNA templates were purified using the QIAquick PCR Purification Kit and quantified using a NanoDrop 1000. RNA was transcribed from 100 nM DNA templates in a buffer containing 40 mM Tris-HCl (pH 8), 8 mM MgCl_2_, 5 mM DTT, 2 mM spermidine, 1 mM rNTPs, 0.01% Tween-20 and 1 U/μl T7 RNA polymerase at 37°C for 60 min. Fluorescence analysis of BRB mixed with the RNA transcripts was performed using the same process used to evaluate the sorted RNAs.

### Binding constant analyses

Variants of RNA duplexes were prepared by annealing the sense and antisense strands listed in Supplementary Table S3 at a 1:1 molar ratio. Varying concentrations (0–4 μM) of the RNA variants were mixed with BRB (50 nM) in a buffer containing 50 mM MES-LiOH (pH 7), 0.5 mM MgCl_2_, 0.1% DMSO and 0.01% Tween-20 in the presence of the indicated concentrations of KCl, and subsequently incubated at 70°C for 5 min and cooled to 25°C at 1°C min^−1^. The fluorescence intensity of BRB in the mixture was measured in a 384-well plate for a small volume sample (384-Well Small Volume HiBase; Greiner Bio-One Co. Ltd., Tokyo, Japan) using InfiniteM200 PRO at 365 nm excitation and 550 nm emission after incubation at 25°C for 60 min. The observed binding constant (*K*_obs_) between BRB and RNA at 25°C was calculated from changes in the fluorescence signals depending on the RNA concentration according to equation 1 in a bellow, assuming 1:1 binding stoichiometry.

(1) $$\begin{eqnarray*}F &=& {F_{{\rm initial}}}\ + \ \left( {\frac{{{F_{{\rm final}}} - {F_{{\rm initial}}}}}{{2\ \times \left[ {BRB} \right]}}} \right)\Bigg\lbrace\left([{BRB}] + [{RNA}] + \frac{1}{{K_{{\rm obs}}}}\right)\nonumber\\ &&-{\sqrt {\left([BRB]+[RNA] +\frac{1}{{K_{{\rm obs}}}} \right)^2-4{\times}[BRB]{\times}[RNA]}}\Bigg\rbrace\end{eqnarray*}$$

where$\ F$ is the fluorescence intensity of BRB at each concentration of RNA, ${F_{{\rm initial}}}$ is the fluorescence intensity of BRB in the absence of RNA, ${F_{{\rm Final}}}$ is the fluorescence intensity of BRB when all BRB binds to RNA, and $[ {RNA} ]$ and $[ {BRB} ]$ are the concentrations of RNA and BRB, respectively.

### NMR spectroscopy

NMR data of RNA and its complex with BRB were collected on an Agilent VNMRS 800 MHz NMR spectrometer equipped with a cryogenic probe. RNA oligonucleotides were dissolved in 10 mM Na_2_HPO_4_ buffer (pH 7) with the addition of 100 mM NaCl, 1 mM EDTA and 10% ^2^H_2_O. The final RNA concentration of individual strands in 300 μl was 300 μM. 1D ^1^H and 2D NOESY and TOCSY spectra were acquired at 25°C utilizing excitation sculpting solvent suppression. The processing and assignment of spectra were performed using NMRPipe and CcpNmr software.

### Molecular dynamics

The molecular dynamics (MD) were calculated in AMBER 20 software using the ff99OL3 force field. The force-field parameters for BRB were derived from the RESP ESP Charge Derive Server. The initial linear structures of oligonucleotides, created with the LEAP module of AMBER, were subjected to MD with a gradual turning on of the restraints until a duplex was obtained. Subsequently, one BRB molecule was added 20 Å off the central axis of the duplex and used as a starting structure. A total of 100 structures were obtained in 1-ns restrained, simulated annealing simulations using the Born implicit solvent model with random starting velocities. In the first 200 ps of simulated annealing, the temperature was kept at 1000 K, followed by slow cooling in the next 400 ps down to 300 K and to 0 K in the last 400 ps. The force constants were 20 kcal·mol^−1^Å^−2^ for hydrogen bonds, 20 kcal·mol^−1^Å^−2^ for NOE distance restraints, 100 kcal·mol^−1^rad^−2^ for backbone and 100 kcal·mol^−1^rad^−2^ for the χ torsion angle. All cross-peaks in imino-aromatic, imino-imino, amino-imino and amino-aromatic regions were classified as strong (2.5 Å), medium (3.5 Å) or weak (4.5 Å), with restraint ranges of ±1–1.5 Å. The dihedral angle *χ* was restrained to an anti range between 90° and 270° for all nucleotides. The restraints for the backbone dihedral angles included *α* (-120° to 120°), *β* (150° to 210°), *γ* (30° to 90°), *ϵ* (170°-300°) and *ζ* (-120° to 120°). Bulged nucleobases were excluded from backbone torsion angle restraints. Depending on the sugar pucker (derived from the TOCSY spectra), the *δ* torsion angle was restrained to the C3′-endo range between 55° and 115° or to the C2′-endo range between 130° and 190°. Ten structures were selected based on the lowest energy and subjected to energy minimization with a maximum of 1000 steps. Molecules were visualized using Chimera software.

### Isothermal titration calorimetry (ITC) studies

The thermodynamic parameters for the interaction between RNA and ligands were determined using MicroCal iTC 200 (Malvern Panalytical, Malvern, United Kingdom). A 10 mM stock solution of ligands in DMSO was diluted 1000-fold into a buffer containing 10 mM Na_2_HPO_4_ (pH 7), 100 mM NaCl and 1 mM EDTA to a concentration of 10 μM and placed in an ITC cell. RNA sample (100 μM) dissolved in a buffer containing 10 mM Na_2_HPO_4_ (pH 7), 100 mM NaCl, 1 mM EDTA and 0.1% DMSO was prepared by incubating at 70°C for 5 min, cooled to 25°C at 1°C min^−1^ and collected in an ITC syringe. The injection of RNA (2 μl) was performed 19 times at time intervals of 150 s. The thermogram obtained was normalized by subtracting that obtained by RNA titration into the buffer without ligand and fitted using MicroCal software following a single binding site model.

### Melting analyses of RNA oligonucleotides

The melting of RNA oligonucleotides (2 μM) was measured by absorption at 260 nm in a buffer containing 10 mM Na_2_HPO_4_ (pH 7), 100 mM NaCl, 1 mM EDTA and 0.1% DMSO in the absence or presence of 10 μM ligand with a heating rate of 0.5°C min^–1^ using a Shimadzu-1800 UV/Vis spectrophotometer equipped with a temperature controller. The thermodynamic parameters were calculated from the melting curves following the two-state melting transition model of oligonucleotides (37–39).

## RESULTS AND DISCUSSION

### Selection of RNAs interacting with BRB by using R-CAMPs

In our previous strategy for the optimization of RNA aptamers using R-CAMPs, we used an artificially designed library containing randomized mutations at the region, which contribute to the interaction between RNA and small molecules, based on the tertiary structure of the aptamers (34,35). However, the types of secondary and tertiary structure motifs preferred by BRB remain unclear. One of the advantages of RNA optimization using R-CAMPs is that it allows constructing R-CAMPs, which have millions of sequence variations, from any kind of sequence library. Thus, in this study, we used total RNA transcripts derived from HeLa cells as the initial library to screen natural RNA targets that potentially interact with BRB (Supplementary Figure S1). HeLa is one of the human cell lines widely used in biological researches. The total RNA of HeLa would be a suitable library for the RNA screening, because transcriptomic data of HeLa cells is available (40). Since rRNAs, which account for the majority of RNA transcripts, potentially cause bias and lack of variation in the initial library, they were removed from the total RNA. The first step in constructing R-CAMPs involved the preparation of a template DNA library and the subsequent individual amplification and immobilization of the DNA on microsphere particles using emulsion PCR. The DNA library obtained after reverse transcription of the fragmented RNA library was immobilized on microsphere particles using a usual experimental procedure for next-generation sequencing. R-CAMPs, which individually display clones of DNA and RNA derived from the library, were prepared as previously reported (34,35). Detailed processes for preparing R-CAMPs are described in ‘Materials and Methods’ section and supporting information. The resulting R-CAMPs were then mixed with BRB to select RNAs interacting with BRB.

**Figure 1.**
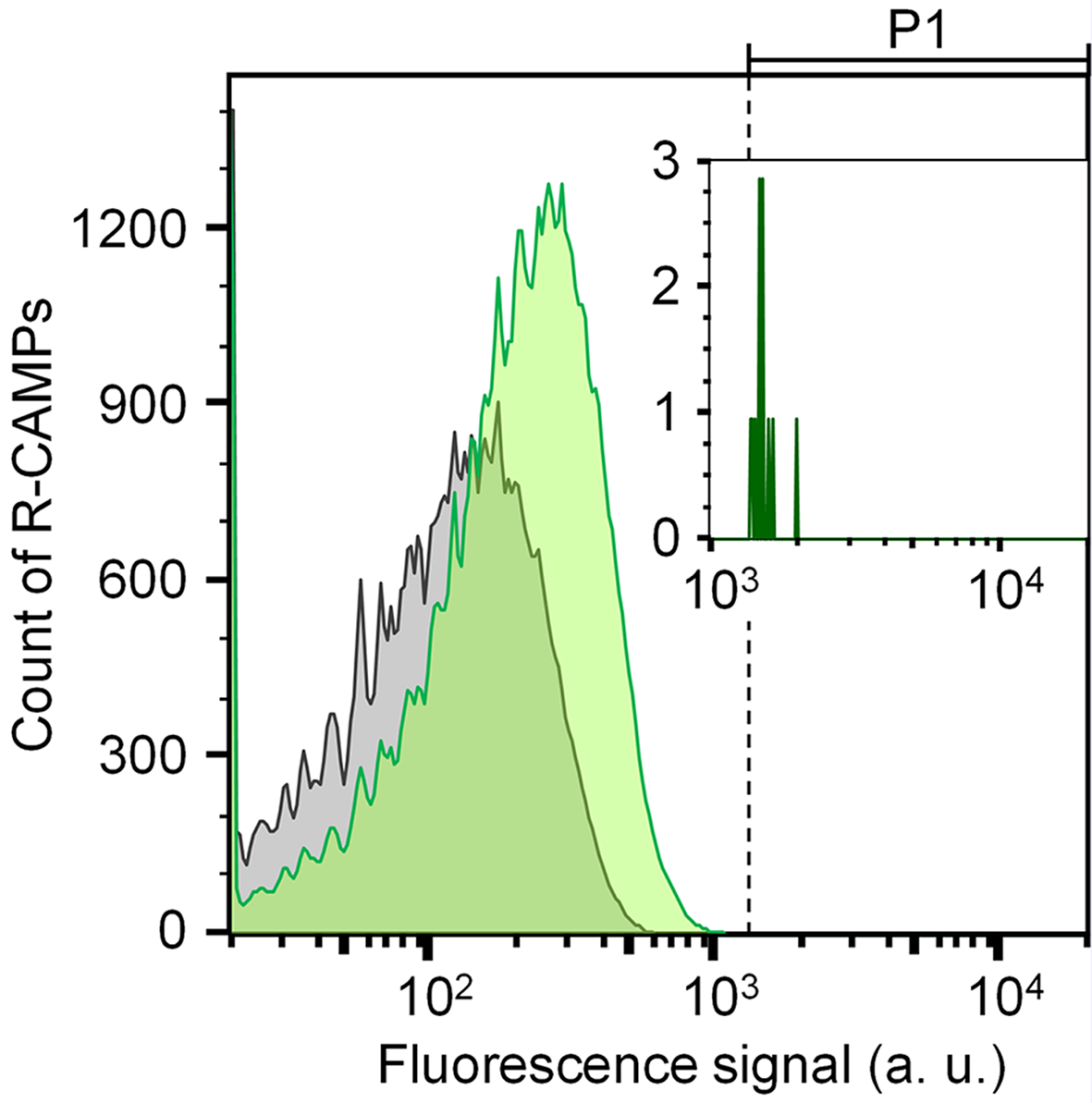
The fluorescence signals of BRB on R-CAMPs. R-CAMPs and control particles were prepared with (green) and without (grey) transcription reaction, respectively, and analysed with BRB (1 μM) in a buffer containing 50 mM MES-Li0H (pH 7), 100 mM KCl, 0.5 mM MgCl_2_, 0.1% DMSO and 0.01% Tween-20. The fluorescence intensities were analysed using 488-nm excitation laser and 530/30-nm bandpass filter. The P1 gate for the selection of R-CAMPs is shown above the graph. R-CAMPs signals observed in the range of the P1 gate are enlarged inset. Twelve R-CAMPs and no control particles were observed in the P1 gate.

Figure 1 shows the fluorescence signals of R-CAMPs analysed by FACS. Among 100 000 particles counted, those distinguished as single particle (78663 R-CAMPs and 76854 control particles) were included in the figure. Overall, the fluorescence signals of BRB mixed with R-CAMPs were higher than those mixed with control particles, which only displayed template DNA strands without a transcription reaction. As described above, since BRB emits fluorescence in the state of a complex with nucleic acids, R-CAMPs with a strong fluorescence are considered to display RNAs interacting with BRB. To select the RNAs that BRB preferentially binds, R-CAMPs showing an arbitrary unit of fluorescence signal over 1300 (P1 region in Figure 1) were sorted. Here, approximately 1.7 million particles flowed into FACS, and 71 particles were sorted one-by-one into a 96-well plate. The number of R-CAMPs supplied for sorting did not completely cover the validation of the transcriptome RNAs. However, considering that R-CAMPs were sorted with an abundance ratio of <0.005%, it was expected that the RNAs with which BRB selectively bound could be extracted.

### Evaluation of sequences and secondary structures of RNAs interacting with BRB

Since each of the sorted R-CAMPs displayed both DNA and RNA clones on the surface, PCR amplification was performed to prepare the cloned template DNA. Unfortunately, not all well positions, where the R-CAMPs were sorted, showed an amplification profile when the amplification process was monitored by quantitative real-time PCR (Supplementary Figure S2A). A total of 42 well positions with fluorescence signals over 10 000 arbitrary units after 15 cycles of PCR contained sorted R-CAMP; the fluorescence increase in the other well positions was considerably delayed (Supplementary Figure S2B). Some of the sorted droplets containing the single particle either did not enter the well of the 96-well plate or landed in a position where they did not soak in the reaction solution for PCR.

All 42 RNAs, which were displayed on the sorted R-CAMPs, were individually transcribed from the amplified template DNA and subsequently mixed with BRB to verify the fluorescence signal of BRB in solution (Supplementary Figure S3). All the samples showed fluorescence signals considerably higher than the signal of BRB mixed with a solution of transcription reaction without DNA template, suggesting that RNAs interacting with BRB were successfully sorted. Among the samples, eight samples showed fluorescence signals significantly higher than other samples. These RNAs were expected to interact with BRB with a higher affinity than other selected RNAs.

The RNA sequences of the eight samples were analysed and verified to determine which RNA transcripts encoded the sequences (Supplementary Table S4). As a result of sequence alignment using the Basic Local Alignment Search Tool (BLAST), all RNAs were assigned to a transcriptome or genome sequence database. The assigned regions included mature mRNAs, primary transcript with introns, rRNAs, chromosomes and mitochondrial genome. However, the sequence inserted between the designed primers was found to partially match the database, and contained RNA sequences of unknown origin. The part where the sequences did not match with the database was hypothesized to originate from another RNA fragment or a random region in the oligonucleotide primers during the ligation reaction to create the cDNA library (41). Due to this problem, it was difficult to determine natural RNA transcripts that could represent as targets of BRB. Thus, the RNA motif was narrowed down to those with which BRB interacted.

**Figure 2.**
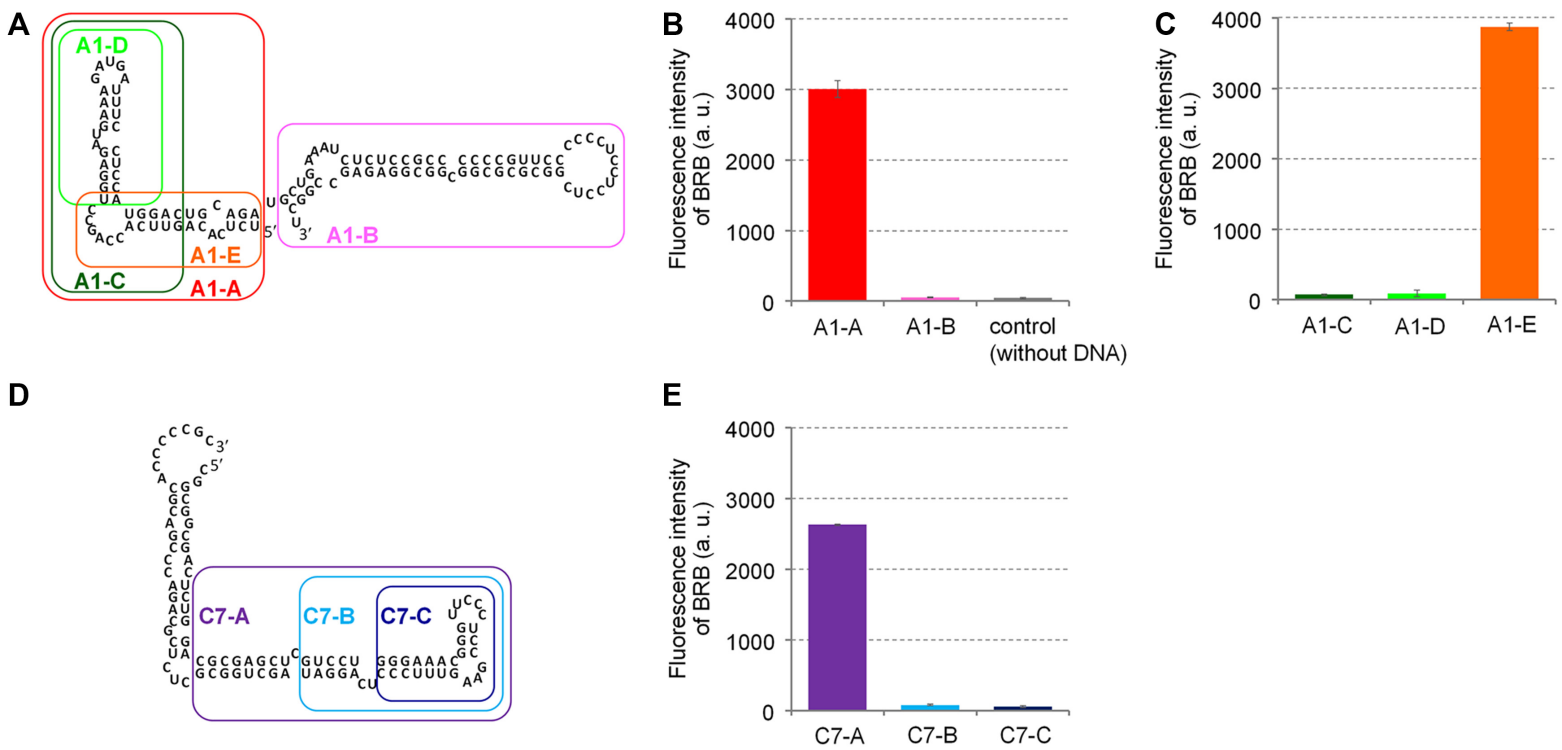
Fluorescence signals of BRB mixed with RNA fragments. (**A**) Design of RNA fragments derived from A1 RNA. (**B** and **C**) Fluorescence signals of BRB mixed with the RNA fragments derived from A1 RNA. (**D**) Design of RNA fragments derived from C7 RNA. (**E**) Fluorescence signals of BRB mixed with the RNA fragments derived from C7 RNA. RNA fragments were prepared by *in vitro* transcription with consecutive two guanine nucleotides at 5′ terminus (Supplementary Table S5). In B, C and E, the RNA transcripts were directly diluted into a buffer containing 50 mM MES-LiOH (pH 7), 100 mM KCl, 0.5 mM MgCl_2_, 1 μM BRB, 0.1% DMSO and 0.01% Tween-20. The fluorescence intensities of BRB in the reaction mixtures were measured using 365 nm excitation and 550 nm emission after incubation at 25°C for 60 min. The reaction buffer contains Tris, DTT, rNTP, T7 RNA polymerase and DNase derived from the dilution of the transcription buffer.

Four RNA samples sorted into the well positions A1, A10, C7 and C10, which showed a particularly strong fluorescence of BRB (Supplementary Figure S3), were selected to identify the RNA region important for the BRB interaction. The RNA sequences shown in Supplementary Table S4 were transcribed from individually synthesized template DNA, and the fluorescence signals of BRB mixed with the transcripts were evaluated (Supplementary Figure S4). Unexpectedly, the two RNAs, A10 and C10, did not increase BRB fluorescence. The initially sorted RNAs contained 5′ and 3′ flanking sequences in addition to the inserted sequence shown in Supplementary Table S4. The fluorescence signals observed with RNA transcripts of A10 and C10 (Supplementary Figure S3) may have been caused by interactions between BRB and the region formed between the inserted and flanking regions. Thus, A1 and C7 RNAs were further separated into short RNA fragments (Supplementary Table S5) to identify the structural units of the BRB interaction. A1 RNA was first separated into two secondary structure units, A1-A and A1-B (Figure 2A). As per the procedure in Supplementary Figure S4, the fluorescence signal of BRB was evaluated by mixing the RNA transcripts. The results showed that BRB mixed with A1-A showed high levels of fluorescence, whereas that mixed with A1-B showed a signal as low as the control sample mixed with the solution for transcription reaction without DNA template (Figure 2B). Then, A1-A was further separated into three shorter units, A1-C, A1-D and A1-E (Figure 2A), and the fluorescence signal of BRB was evaluated in the same manner (Figure 2C). Only A1-E was found to increase the fluorescence signal, suggesting that BRB interacted with a simple hairpin RNA containing an internal loop (Figure 2A). In the case of C7 RNA, the sequence was predicted to form continuous secondary structural units. Thus, three RNA variants, C7-A, C7-B and C7-C, were prepared by the stepwise removal of the secondary structure unit (Figure 2D). As a result, the high levels of fluorescence of BRB observed upon mixing with C7-A were completely lost when the RNA was shortened to C7-B and C7-C (Figure 2E). These results suggest that a single cytosine bulge (C-bulge), which was included in C7-A but excluded in C7-B and C7-C, most likely interacted with BRB. This is supported by the results obtained from the A1 RNA variants, because the internal loop region in A1-E likely form a single C-bulge when A and C in the internal loop form a mismatch base pair (42–44). On the other hand, A1-B did not show BRB fluorescence (Figure 2B) despite containing a single C-bulge (Figure 2A). These results suggest that BRB interacts with RNA regions containing a C-bulge with a degree of sequence selectivity.

### Optimized core unit for BRB interaction

**Figure 3.**
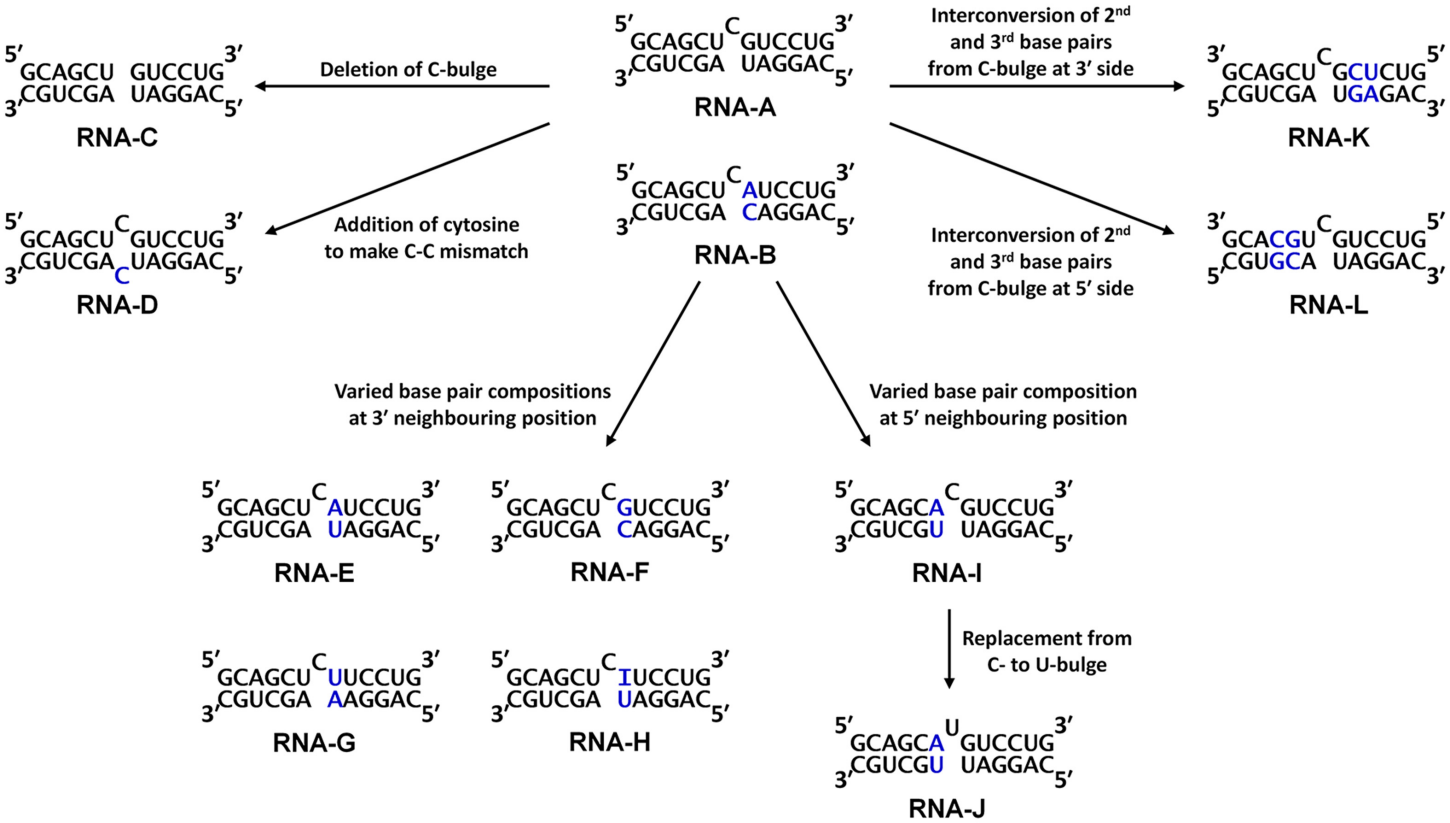
Sequence designs of bulged RNA duplexes. Nucleobases different from RNA-A are shown in blue.

**Figure 4.**
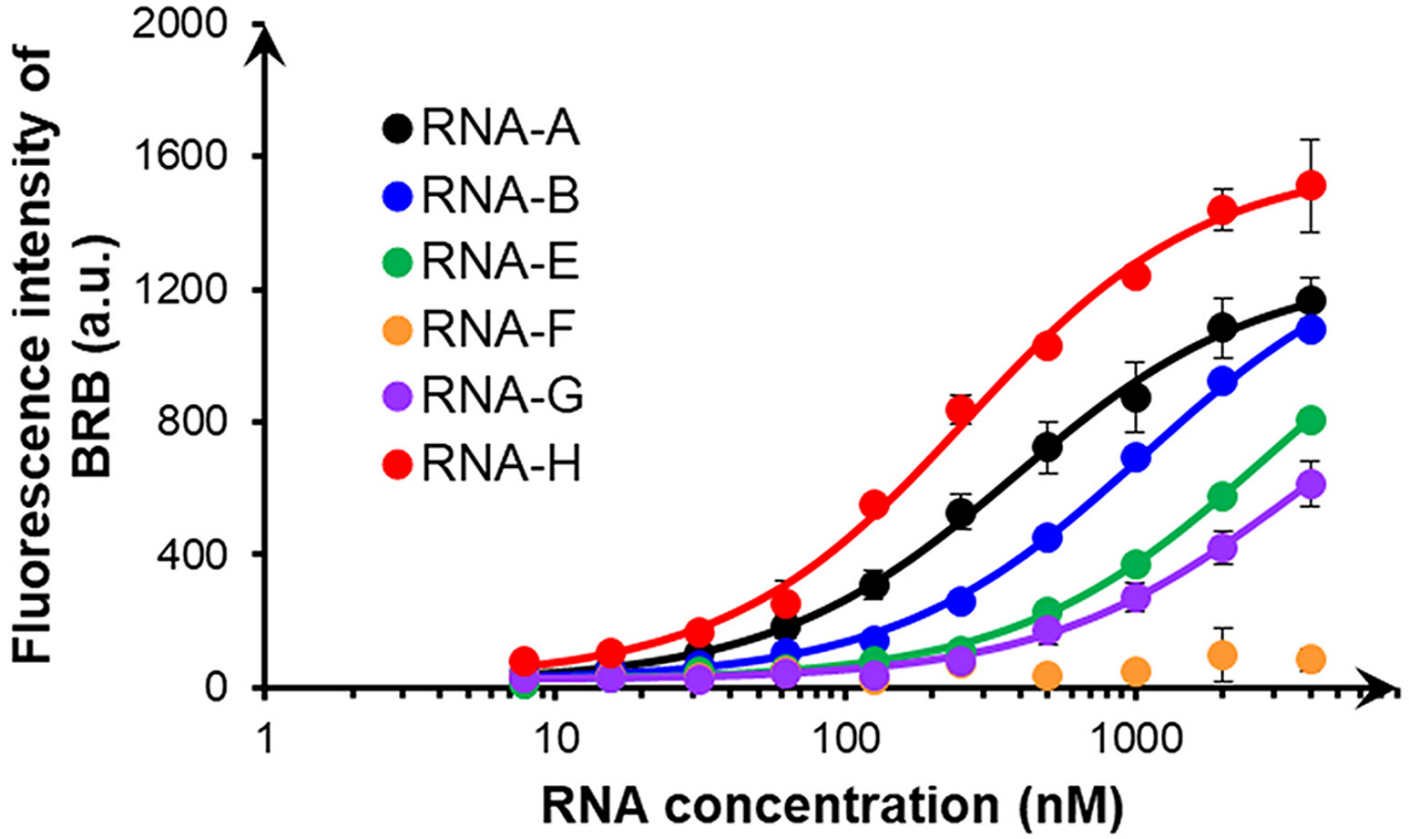
Fluorescence intensities of BRB mixed with RNA variants. BRB (50 nM) was mixed with various concentrations of RNA-A (black), RNA-B (blue), RNA-E (green), RNA-F (orange), RNA-G (purple) or RNA-H (red) in a buffer containing 50 mM MES-LiOH (pH 7), 0.5 mM MgCl_2_, 100 mM KCl, 0.1% DMSO and 0.01% Tween-20. The fluorescence signals of BRB were measured at 25°C after 60-min incubation using 365 nm excitation and 550 nm emission. Values and errors represent the average ± S.D. of triplicated experiments.

To quantitatively verify the sequence selectivity of BRB, variants of the bulged RNA duplex, some of which are complementary duplex and mismatched duplex, were systematically designed (Figure 3). RNA-A and RNA-B were constructed to mimic the secondary structure elements in C7-A and A1-E RNAs, respectively. RNA-A had a single C-bulge with U-A Watson–Crick and G-U wobble base pairs at the 5′ and 3′ neighbouring positions of the bulge, respectively, while RNA-B had an internal loop consisting of CA and C on sense and antisense strands, respectively. RNA-B is also considered to have a single C-bulge with U-A Watson–Crick and C-A mismatch base pairs at the 5′ and 3′ neighbouring positions, respectively. These RNAs increased the fluorescence signals of BRB in a concentration-dependent manner (Figure 4), with an observed association constant at 25°C (*K*_obs_) of 2.79 and 0.98 × 10^6^ M^−1^ for RNA-A and RNA-B, respectively (Table 1). These values were considerably higher than the affinity value between tRNA and BRB, in which the observed binding constant was approximately 10^5^ M^−1^ (32). Although BRB was reported to interact with the RNA duplex, the binding affinities were around 10^4^ M^−1^ (29). Consistent with the literature (29), BRB did not show any fluorescence change with RNA-C (Supplementary Figure S5), which is a complementary duplex with a deletion in the cytosine bulge of RNA-A, at experimental RNA concentrations (0–4 μM). These results suggest that, despite being simple RNA duplexes with small bulge and internal loop regions, RNA-A and RNA-B have markedly high binding affinities toward BRB. By contrast, RNA-D, which has a single C-C mismatch, showed no interaction with BRB (Supplementary Figure S5 and Table 1), suggesting that a single nucleotide bulge or an asymmetric internal loop is important for the BRB interaction.

**Table 1.** *K*_obs_ values between BRB and RNA variants at 25°C

RNA	*K*_obs_ (× 10^6^ M^−1^)^a^	RNA	*K*_obs_ (× 10^6^ M^−1^)^a^
A	2.79 ± 0.64	G	0.29 ± 0.01
B	0.98 ± 0.25	H	4.27 ± 0.88
C	n.d.^b^	I	0.43 ± 0.06
D	n.d.^b^	J	n.d.^b^
E	0.36 ± 0.04	K	2.37 ± 0.43
F	n.d.^b^	L	1.88 ± 0.18

^a^Values are average ± S.D. of triplicated experiments.

^b^Affinity was too low to obtain accurate *K*_obs_ value.

The difference in the *K*_obs_ values between RNA-A and RNA-B suggested that the mismatch base pair at the 3′-neighbouring position of the C-bulge is disfavoured for the interaction. Thus, RNA-E to RNA-H were designed with various base pair compositions, including a wobble base pair of inosine (I) and uracil in RNA-H, at the 3′ neighbouring position (Figure 3). The fluorescence intensities of BRB with increasing concentrations of these RNAs are shown in Figure 4. Interestingly, the insertion of Watson–Crick base pairs reduced the *K*_obs_ values. In particular, when the base pair was replaced by a G-C Watson–Crick base pair as in RNA-F, the BRB interaction was lost. The replacement to the A-U or U-A Watson–Crick base pairs as in RNA-E or RNA-G, respectively, decreased the binding affinity by approximately 8- or 10-fold, respectively, resulting in *K*_obs_ values lower than RNA-B (Table 1). In contrast, the insertion of the I-U wobble base pair as in RNA-H increased the *K*_obs_ value compared to RNA-A (Table 1). Thus, the wobble base pair composition at the 3′-end of the bulge may improve its interaction with BRB. A–C mismatch in RNA-B potentially forms a base pair with the wobble base pair configuration, especially when the nitrogen of adenine at the N1 position is protonated (42–44), and thus likely showed larger *K*_obs_ values than those consisting of Watson–Crick base pairs.

In the case of base pair composition at the 5′-neighbouring position of the C-bulge, the replacement of a U-A Watson–Crick base pair in RNA-A by a A-U Watson–Crick base pair in RNA-I reduced the *K*_obs_ value by approximately 6.5-fold (Figures 3 and Supplementary Figure S6, and Table 1). This suggests that the base pair composition at the 5′-end also plays a vital role in its interaction with BRB. Additionally, the importance of cytosine at the bulge was also explored by comparing the *K*_obs_ values between RNA-I and RNA-J, where the cytosine at the bulge was replaced by uracil (Figure 3). The binding affinity of RNA-J towards BRB was found to be completely lost in the experimental range (Supplementary Figure S6).

Based on the relatively small chemical structure of BRB, the C-bulge and base pairs at both the 5′- and 3′-end are likely to be minimum and sufficient for the formation of a BRB binding site. To confirm this, the base pair compositions of RNA-A were altered at the second and third positions from the C-bulge on both the 3′- and 5′-ends (Figure 3). Consequently, the *K*_obs_ values obtained using RNA-K (2.37 × 10^6^ M^−1^), with exchanged base pairs at the 3′-end, and RNA-L (1.88 × 10^6^ M^−1^), with exchanged base pairs at the 5′-end, were comparable with those obtained by RNA-A (Supplementary Figure S6 and Table 1). The preferential binding motif of BRB was found to be the C-bulge with U-A and G-U neighbouring base pairs at 5′ and 3′, respectively. Although the binding site consists of five nucleobases with a single bulged nucleobase, the affinity was higher than other chemical compounds that target RNA with more bulged nucleobases such as transactivation response (TAR) RNA and Rev response element (RRE) RNA in human immunodeficiency virus (5).

### NMR structural analyses of complex state between BRB and RNA-A

**Figure 5.**
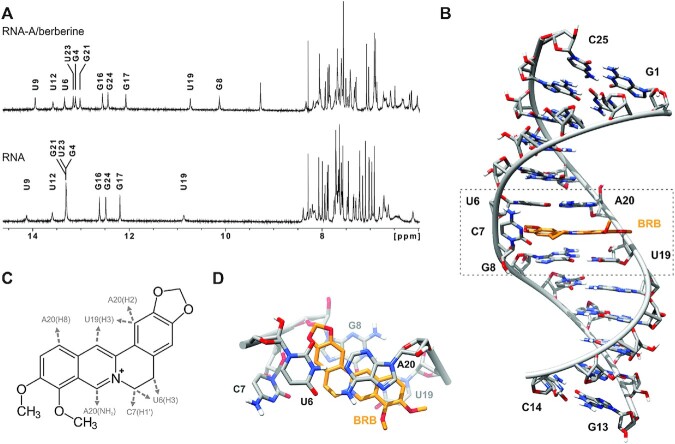
(**A**) Comparison of aromatic and imino regions of 1D ^1^H NMR spectra of the RNA-A and RNA-A/BRB complex. Imino proton resonances are assigned. (**B**) Representative high resolution structure of the RNA-A/BRB complex. (**C**) NOE contacts between BRB and RNA protons. Arrows indicate the point of contact with nucleotides. (**D**) Top view of the bulged region of the complex showing stacking of BRB with adjacent nucleobases.

As RNA-A showed the highest binding affinity to BRB, NMR analysis was performed for RNA-A and its complexation with BRB to further explore their interactions. RNA-A solution in the absence of BRB was found to exhibit imino proton signals characteristic of Watson–Crick base pairs (Figure 5A). However, no imino resonances were observed for terminal nucleotides G1 and G13 (Figure 5B), which was expected due to efficient exchange with the solvent. In addition to the terminal nucleotides, imino resonances of U6 and G8 were also absent, while those of U9 and U19 were significantly broadened. The absence and broadening of imino signals corresponding to nucleotides close to the C-bulge (i.e. C7) suggested that the bulge and neighbouring base pairs were dynamic compared to the remainder of the duplex.

The addition of BRB to the RNA-A solution at a 1:1 molar ratio resulted in sharp imino signals for the U9 and U19 nucleobases, as well as increased intensities. In addition, imino resonances of the U6 and G8 appeared in the spectrum (Figure 5A). These results indicate that BRB interacted with RNA-A at a site close to the C-bulge, as suggested by fluorescence titration, and induced formation of neighbouring U-A and G-U base pairs. To identify the chemical groups/moiety of BRB involved in its interaction with RNA-A, BRB resonances were assigned via analysis of NOESY and TOCSY spectra of the RNA-A/BRB complex. A single set of NMR signals for BRB, with chemical shifts deviating considerably from signals of an aqueous BRB solution, was observed in the NMR spectra of the complex (Supplementary Table S6). Interestingly, several NOE contacts were identified between BRB and RNA protons (Figure 5C). As expected, U6, C7, U19 and A20 nucleotides were found to be involved in NOE contacts with BRB, highlighting preferential binding of BRB to the site of the C-bulge. Based on NMR data and a simple simulated annealing protocol, a high-resolution model of the RNA-A/BRB complex was constructed. A converged set of structures was obtained, of which ten were chosen for further refinement (Supplementary Table S7).

The structural model indicated that BRB intercalates between U6-A20 and G8-U19 base pairs adjacent to the C-bulge (Figure 5B). In addition, the RNA was found to adopt an A-type double helix with all nucleotides in the anti-conformation with N-type sugar puckers, except for C7 and A20, which exhibited S-type sugar puckers. The bulged C7 was found to flip out of the helix into the major groove and did not rotate freely because of hydrogen bonding between the amino group of C7 and the phosphate group of U6. This is supported by NMR data, with the bonded and non-bonded amino proton resonances of C7 at 7.88 and 6.86 ppm, respectively (Supplementary Figure S7). Although C7 did not directly interact with BRB, the replacement of the C-bulge in RNA-I to the U-bulge in RNA-J reduced the BRB affinity (Table 1). It is considered that C7 supports the optimal backbone structure via the formation of a hydrogen bond with the phosphate. BRB was co-planar to the U6-A20, and G8-U19 base pairs, and more prominent stacking was observed with the G8-U19 base pair compared to the U6-A20 base pair (Figure 5D). Although molecules, which intercalate into nucleic acids, generally do not show sequence selectivity (45,46), BRB was found to exhibit optimal binding to a site consisting of a C-bulge with U-A and G-U neighbouring base pairs. In particular, the wobble base pair configuration was found to be important for binding (Table 1). The glycosidic bond angles of the U nucleobase in the G-U wobble base pair were larger than those of the conventional Watson–Crick base pair (65° in G-U and 54° in G-C/A-U) (47,48), resulting in a wider major groove, which may be useful for ligand interactions (49–51). The wider major groove and the orientation of the wobble base pair, in which uracil nucleobase slides into the major groove side, are considered favourable for enhancing the stacking interaction that results in sequence preference.

### Effect of electrostatic interaction on the BRB affinity

**Figure 6.**
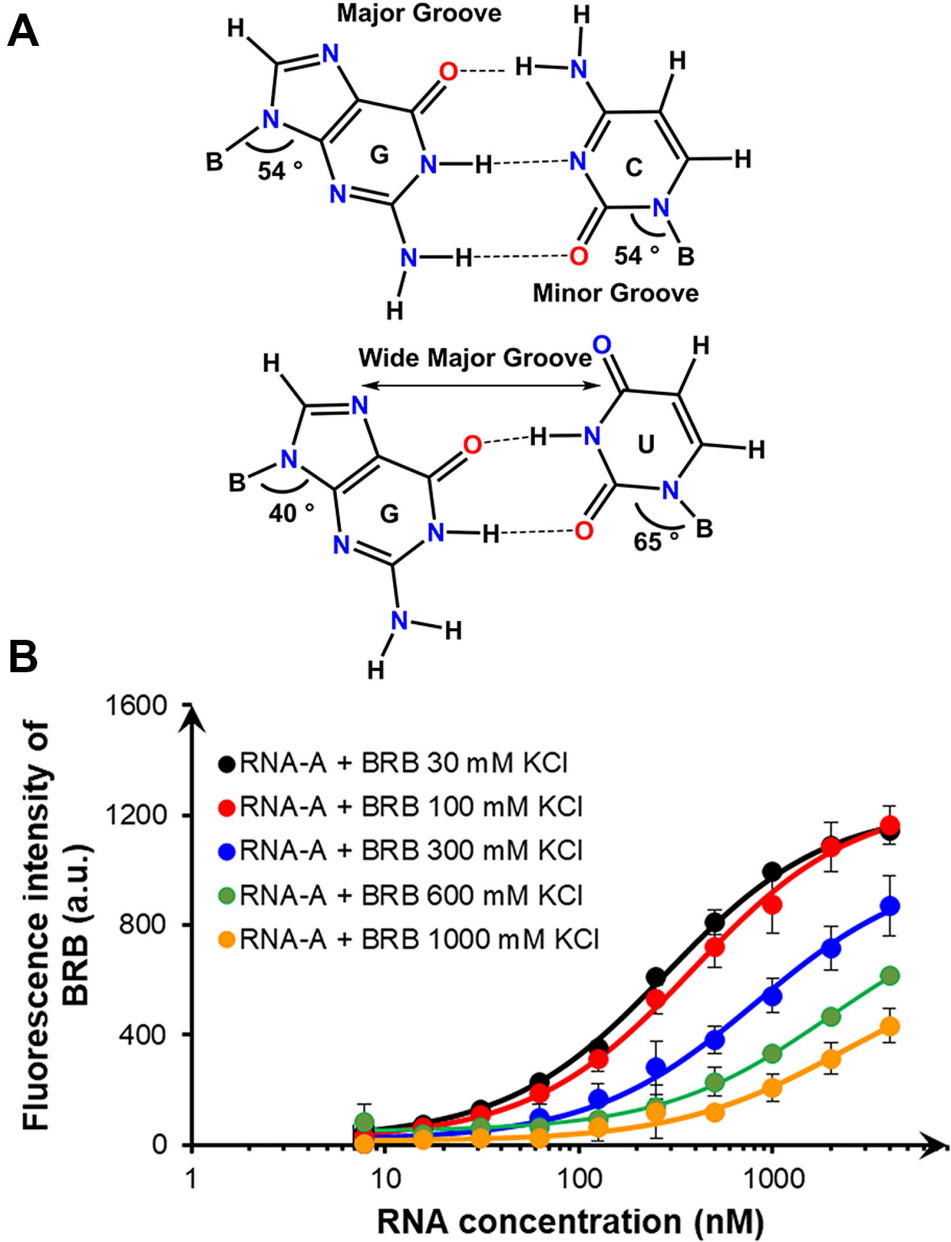
Contribution of electrostatic interaction between BRB and G-U wobble base pair. (**A**) Chemical representation of G-C Watson-Crick and G-U wobble base pairs. (**B**) Effect of KCl concentration on interaction between BRB and RNA-A. BRB (50 nM) was mixed with various concentrations of RNA-A in a buffer containing 50 mM MES-LiOH (pH 7), 0.5 mM MgCl_2_, 0.1% DMSO, and 0.01% Tween-20 in the presence of 30 mM (black), 100 mM (red), 300 mM (blue), 600 mM (green) or 1000 mM (orange) KCl. The fluorescence signals of BRB were measured at 25°C after 60-min incubation using 365 nm excitation and 550 nm emission. Values and errors represent the average ± S.D. of triplicated experiments.

One of the characteristics of the structure of the complex analysed by NMR was that the quaternary nitrogen atom of BRB (Figure 5), which had an electropositive charge, was directed toward the major groove side. As mentioned above, the G-U wobble base pair was found to be well-stacked with BRB. The surface of the G-U wobble base pair at its major groove side contained a series of electronegative atoms (guanine N7, guanine O6 and uracil O4) (Figure 6A), resulting in a distinctive broad electronegative region in the wide major groove (48). The electronegative surface in nucleic acids usually acts as the recognition motif for cationic ligands and metal ions (47,48,52). A similar electrostatic interaction was expected between the quaternary nitrogen atom of BRB and the G-U wobble base pair. To validate these electrostatic interactions, fluorescence titration experiments of BRB with RNA-A were performed in the presence of different concentrations of KCl (Figure 6B). As a result, the binding affinity between BRB and RNA-A was found to decrease 8.7-fold with an increasing concentration of KCl at 25°C: *K*_obs_ value of 3.91 × 10^6^ M^−1^ in the presence of 30 mM KCl decreased to 0.45 × 10^6^ M^−1^ in the presence of 1000 mM KCl (Supplementary Table S8). On the other hand, in the case of RNA-B, which showed no accumulation of electronegative atoms on the major groove side of its A-C mismatch base pair, the *K*_obs_ values were found to decrease by 5.2-fold, from 1.14 × 10^6^ M^−1^ at 30 mM KCl to 0.22 × 10^6^ M^−1^ at 1000 mM KCl (Supplementary Figure S8 and Table S8). As a result, the *K*_obs_ value of RNA-A was found to be more sensitive to KCl concentration than that of RNA-B, demonstrating the contribution of electrostatic interactions on selective interactions between BRB and the G-U wobble base pair. These results also highlight the importance of the electrostatic interaction that would be one of the main courses of higher binding affinity of BRB compared to the other small molecules interacting with bulged RNA helices (5).

### Stabilization of RNA by BRB analogues

**Figure 7.**
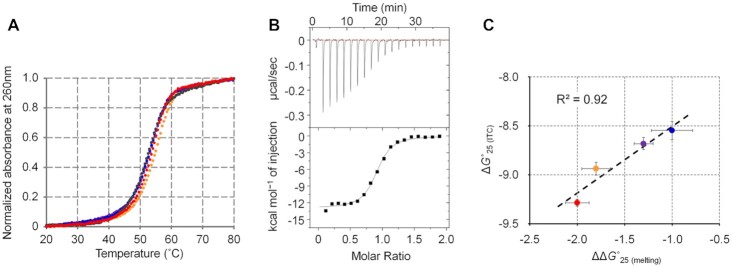
Stabilization of RNA-A depending on binding affinity. (**A**) Normalized UV melting curves of RNA-A (2 μM) in a buffer containing 10 mM Na_2_HPO_4_ (pH 7), 100 mM NaCl, 1 mM EDTA, and 0.1% DMSO in the absence (black) or presence of BRB (red), COP (orange), PAM (purple) or EBRB (blue) at 10 μM. (**B**) ITC thermograms (upper panels) and isotherms (lower panels) for the titration of RNA-A into BRB. RNA-A (100 μM in the syringe) was titrated into BRB (10 μM in the cell) in a buffer containing 10 mM Na_2_HPO_4_ (pH 7), 100 mM NaCl, 1 mM EDTA and 0.1% DMSO at 25°C. The solid line in the lower panel represents the line fitted to a single binding site model. (**C**) Plots between free energy change during the binding reaction between RNA-A and BRB analogues at 25°C obtained from ITC (Δ*G°*_25 (ITC)_) and change in thermodynamic stability of RNA-A provided by BRB analogues (ΔΔ*G°*_25 (melting)_). Colours denote ligands with the same melting profile.

Nucleic acid structures can be stabilized by the specific interaction of small ligands, as demonstrated by studies using aptamers and riboswitches (53,54). The ^1^H NMR spectra revealed the formation of adjacent U-A and G-U base pairs that potentially stabilizes the RNA structure upon BRB binding. The thermodynamic stabilities of RNA-A were investigated by UV melting assay in the absence and presence of BRB at a concentration of 10 μM. BRB analogues (i.e. coptisine (COP), palmatine (PAM) and epiberberine (EBRB) (Table 2)) were also used to investigate the roles of the dimethoxy and dioxolane groups in BRB on RNA stabilization. A buffer containing 10 mM Na_2_HPO_4_, 100 mM NaCl and 1 mM EDTA, which was used as a standard buffer with physiological ionic strength (55), was used for the melting assay. Based on the stock solution of the ligands, the buffer contained 0.1% DMSO. Since the melting profiles showed clear sigmoidal signals (Figure 7A), the thermodynamic parameters were calculated by fitting the melting profiles using the Van’t Hoff equation (Supplementary Table S9) (37–39). Although the rise in the melting temperature (*T*_m_) was not so large for any of the ligands, the ligands were found to significantly enhance the thermodynamic stability of RNA-A at 25°C (Δ*G°*_25 (melting)_) by decreasing the enthalpy change of the melting (Supplementary Table S9). Energetic contributions of the ligands to stabilize RNA-A were calculated as differences in Δ*G°*_25 (melting)_ in the presence of the ligand compared to that in its absence (ΔΔ*G°*_25 (melting)_). The ΔΔ*G°*_25 (melting)_ values for BRB, COP, PAM, and EBRB, were −2.0, −1.8, −1.3 and −1.0 kcal mol^−1^, respectively (Table 2). As the binding core of RNA-A was selected using R-CAMPs using BRB as a target, BRB was expected to have a higher binding affinity than the other analogues.

**Table 2.** Thermodynamic stabilities of RNA-A obtained from UV melting (Δ*G°*_25 (melting)_) and parameters (*K*_A__(ITC)_, Δ*H*_(ITC)_, Δ*S*_(ITC)_ and Δ*G°*_25 (ITC)_) for interaction between RNA-A and BRB analogues obtained by ITC at 25°C

Thermodynamic parameters^a^		BRB 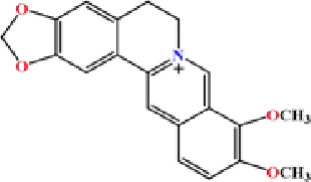	COP 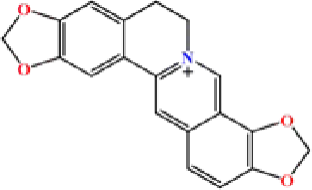	PAM 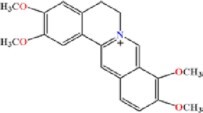	EBRB 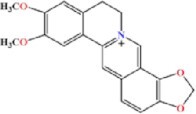
UV melting	ΔΔ*G°*_25, (melting)_ (kcal mol^−1^)	-2.0 ± 0.13	-1.8 ± 0.15	-1.3 ± 0.10	-1.0 ± 0.22
ITC	*n*	0.85 ± 0.03	0.91 ± 0.05	0.88 ± 0.02	0.80 ± 0.01
	*K*_A__(ITC)_ (× 10^6^ M^−1^)	6.47 ± 0.37	3.57 ± 0.31	2.32 ± 0.23	1.85 ± 0.25
	Δ*H*_(ITC)_ (kcal mol^−1^)	−13.3 ± 0.37	−11.8 ± 0.40	−14.4 ± 0.61	−11.6 ± 0.12
	Δ*S*_(ITC)_ (cal mol^−1^ K^−1^)	−13.5 ± 1.32	−9.76 ± 1.40	−19.3 ± 2.23	−10.3 ± 0.64
	Δ*G°*_25 (ITC)_ (kcal mol^−1^)	−9.29 ± 0.03	−8.94 ± 0.06	−8.68 ± 0.06	−8.55 ± 0.09

^a^Values are average ± S.D. of triplicated experiments.

To correlate the binding affinity and the stabilization effect, the interaction between RNA-A and BRB or its analogues was evaluated by isothermal titration calorimetry (ITC) in a buffer containing 10 mM Na_2_HPO_4_ (pH 7), 100 mM NaCl, 1 mM EDTA and 0.1% DMSO, which was the same as the buffer used for the UV melting assay (Figure 7B and Supplementary Figure S9, and Table 2). The results supported a 1:1 binding ratio between ligands and RNA, as revealed by the NMR spectra because a stoichiometry parameter (*n*) obtained from the ITC thermograms was 0.80–0.91. The interaction of all BRB analogues was driven by a favourable enthalpic contribution (negative Δ*H*_(ITC)_ value), likely due to stacking and electrostatic interactions. The order of the equilibrium association constant at 25°C obtained from the ITC profile (*K*_A__(ITC)_) was BRB > COP > PAM > EBRB. These results indicate the importance of the dioxirane ring of BRB to enhance its interaction, despite not having a direct interaction with RNA-A (Figure 5C). The constrained dioxolane ring contributed favourably, reducing the entropic cost during the interaction, since PAM, which has a flexible dimethoxy group instead of the dioxolane, increased the entropic cost (negative Δ*S*_(ITC)_ value) of the interaction. By comparing BRB and COP, the dimethoxy group of BRB on the other side of the dioxolane group was found to make a small contribution to enhance the affinity. Although the dioxolane group showed no direct interaction, it may contribute to the interaction via the rearrangement of the solvation state, such as hydrated water networks (56). A linear correlation was observed between Δ*G°*_25 (ITC)_, which was obtained by ITC using a theoretical equation of Δ*G°*_25 (ITC)_ = Δ*H*_(ITC)_ – *T*Δ*S*_(ITC)_, and ΔΔ*G°*_25 (melting)_ (Figure 7C, and Table 2). The linear correlation suggests that the interaction energy provided by the BRB analogues contributed to stabilizing the RNA in a similar way. Based on the NMR structure, the same heterocyclic part, which is a protoberberine structure at the middle of the ligands, mainly contributed to RNA stabilization through stacking and electrostatic interactions.

In summary, we have demonstrated an integrated approach of physicochemical and structural investigations that enable insight into the interaction between particular RNA motifs and phytochemicals. The RNAs that interacted with medicinally important PCs, such as BRB, was screened using R-CAMPs, and quantitatively explored as an RNA motif of a single C-bulge with U-A and G-U neighbouring base pairs. NMR analysis elucidated the importance of the electrostatic and stacking interactions for stabilizing the RNA that was further demonstrated through the physicochemical analyses. Based on the results, not only BRB but also natural phytochemicals, which are planar heterocycles with electropositive atoms, such as derivatives of protoberberine and quinolinium (57,58), have the potential to stack into RNA helices with some sequence and structure preferences. Thus, a part of the biological activities provided by the phytochemicals is likely mediated by the interaction with RNAs (59–61). The approach to survey RNA structure elements that interact with a target chemical seems opposite to a general approach to develop RNA-targeting therapeutics through screening chemicals targeting specific pathogenic RNA. However, once after some chemical compounds have been screened, it should be necessary to investigate the possibility of the compound interact with other RNAs. Thus, the approach in this study has an important complementary for developing the RNA-targeting therapeutics. In addition, following the RNAs selection, we have performed physicochemical and structural analyses through UV-melting, ITC and NMR studies that provided details of the energetic contribution of the ligand to stabilise the RNA. The basic knowledge obtained in this approach would be useful for expanding phytochemicals to be RNA-targeting chemicals. For example, chemical modifications at the dimethoxy group of BRB, which is exposed to the major groove side of the RNA, would enable enhancement of the binding affinity and the sequence selectivity.

## DATA AVAILABILITY

Atomic coordinates and list of chemical shifts for the RNA-A/berberine structures have been deposited with the Protein Data bank and the Biological Magnetic Resonance Bank with accession numbers PDB ID 7A3Y, BMRB ID 34553.

## Supplementary Material

gkab189_Supplemental_File
