# Supplementary material for: Transcriptome screening followed by integrated physicochemical and structural analyses for investigating RNA-mediated berberine activity
Source: Nucleic Acids Res. 2021 Mar 30;49(15):8449–61. doi: 10.1093/nar/gkab189 (PMC8421223; doi:10.1093/nar/gkab189)
Supplement: gkab189_Supplemental_File [file gkab189_supplemental_file.pdf]

Supporting Information

Transcriptome Screening followed by Integrated Physicochemical and Structural Analyses for Investigating RNA-mediated Berberine Activity

Sagar Satpathi[1,£] Tamaki Endoh[1,£] Peter Podbevšek,[2] Janez Plavec[2,3,4] and Naoki Sugimoto[1,5,*]

[1] Frontier Institute for Biomolecular Engineering Research (FIBER), Konan University, 7-1-20 Minatojima-minamimachi, Kobe, 650-0047, Japan
[2] Slovenian NMR Centre, National Institute of Chemistry, Hajdrihova 19, Ljubljana, SI-1000, Slovenia
[3] EN→FIST Centre of Excellence, Trg OF 13, SI-1000 Ljubljana, Slovenia
[4] Faculty of Chemistry and Chemical Technology, University of Ljubljana, Večna pot 113, p. p. 537, SI-1000 Ljubljana, Slovenia
[5] Graduate School of Frontiers of Innovative Research in Science and Technology (FIRST), Konan University, 7-1-20 Minatojima-minamimachi, Kobe, 650-0047, Japan

[£]Equal contribution from both of the authors.

* To whom correspondence should be addressed. Tel: +81-78-303-1416; Fax: +81-78-303-1495; Email: sugimoto@konan-u.ac.jp

Supporting Methods

Preparation of complementary DNA library from total RNA

A complementary DNA (cDNA) library was prepared from total RNA derived from a human cervical cancer cell line (HeLa), included in the Ion Total RNA-Seq Kit v2 (Thermo Fisher Scientific, Waltham, MA, USA). Ribosomal RNA (rRNA) in the total RNA was removed using the NEBNext rRNA Depletion Kit (New England Biolabs, Ipswich, MA, USA) according to the manufacturer’s protocol. The concentration of the RNA after rRNA depletion was estimated using a NanoDrop 1000 (Thermo Fisher Scientific). According to the manufacturer's protocol, the fragmented cDNA library was prepared using the Ion Total RNA-Seq Kit v2. Briefly, approximately 50 ng of total RNA, in which rRNA was removed, was fragmented by RNase III and purified using the nucleic acid binding beads provided in the kit. DNA adaptors were subsequently ligated to the fragmented RNA, and cDNA was synthesized by reverse transcription. The purified cDNA library was amplified by PCR using an originally designed sense primer (temp-s primer; Table S1), which is indispensable for R-CAMP preparation, and Ion 3′ PCR Primer v2 from Ion Total RNA-Seq Kit v2. The thermal cycle for PCR was set according to the manufacturer’s protocol. From the amplified cDNA fragments, those with a length of approximately 200–250 base pairs were extracted using E-Gel SizeSelect II Agarose Gel (Thermo Fisher Scientific). The concentration of the cDNA fragments after size selection was quantitated using the Mx3000P qPCR system (Agilent, Santa Clara, CA, USA) using a set of A-primer and B-trP1 primer (Table S1) compared with a known concentration of a standard DNA fragment.

Preparation of microsphere particles displaying single-stranded DNAs

The fragmented cDNA library (total of 200 amol) was mixed with the microsphere particles (MPs) provided in Ion PGM Hi-Q View OT2 Kit (Thermo Fisher Scientific). Emulsion PCR and purification of MPs, each of which immobilizes clones of single-stranded DNA templates derived from the cDNA library, were performed using the Ion OneTouch 2 system (Thermo Fisher Scientific) according to the manufacturer’s protocol of Ion PGM Hi-Q View OT2 Kit.

Table S1. Oligonucleotides for preparing cDNA library and R-CAMPs

Oligonucleotide	Sequence
temp-s primer	CCATCTCATCCCTGCGTGTCTCCGACTCAGTAATACGACTCACTATAG GGCGAATCAGCCGATTACTAGAGCAGAAACGGCCAAGGCG
A-primer	CCATCTCATCCCTGCGTGTCTCCGACTCAG
B-trP1 primer	CTATCCCCTGTGTGCCTTGGCAGTCTCAGCCTCTCTATGGGCAGTCG GTGAT
capture primer [a]	GCTCTAGTAATCGGCTGATTCGCCC-link-CAGTAATACGACTCACTATAGGG
capture primer (labeled with AF647) [a]	AF647-GCTCTAGTAATCGGCTGATTCGCCC-link-CAGTAATACGACTCACTATAGGG

a. “AF647” indicates Alexa Fluor 647 dye, and “-link-” indicates oligo ethylene glycol linker.

Table S2 Oligonucleotides for preparing DNA templates for RNA transcription

RNA	Reaction		Sequence
A1	primer extension	sense	TAATACGACTCACTATAGGTCTCACAGTTCACCAGCCTGGAGATGA AAGATGATTTCCTCCATGGACTGCAGATGCTGAA
		antisense	AGCCGGCTCTCCGCCGCCGCGCGCCGAGGAGGAGGGGGGAACG GGGGGCGGAGAGATTTCAGCATCTGCAGTCCATGG
A10	PCR	sense	TAATACGACTCACTATAGGTGATAGCTCTTTCTCGATTCCGTGGGT GGTGGTGCATGGCCGTT
		template	ATTACACAGTGCTTTAAAAGTGACTGGTATGAATCATCCTGTCACA TCACAAGAAGAGAACGGCCATGCACCACCACC
		antisense	GATGCTAAGATGTTTCTAAGGACTCTGCTCATTACACAGTGCTTTA AAAGTGACTG
C7	primer extension	sense	TAATACGACTCACTATAGGCGGCGGCGACTCTGGACGCGAGCTCG TCCTGGGAAACGGGTTCCCTCCGA
		antisense	GCGGGGGTGCGTCGGGTCTGCGAGAGCGCCAGCTATCCTGAGG GAAACTTCGGAGGGAACCCGTTTCCC
C10	primer extension	sense	TAATACGACTCACTATAGGTCTCTATTTTACCCTCCTACAAGCCTCA GAGTACTTCGAGTC
		antisense	CTGTGGCTACAAAAAATGTTGAGCCGTAGATGCCGTCGGAAATGG TGAAGGGAGACTCGAAGTACTCTGAGGC
A1-A	primer extension	sense [a]	TAATACGACTCACTATAGG
		antisense	TCTGCAGTCCATGGAGGAAATCATCTTTCATCTCCAGGCTGGTGAA CTGTGAGACCTATAGTGAGTCGTATTA
A1-B	primer extension	sense [a]	TAATACGACTCACTATAGG
		antisense	GCCGGCTCTCCGCCGCCGCGCGCCGAGGAGGAGGGGGGAACGG GGGGCGGAGAGATTTCAGCCTATAGTGAGTCGTATTA
A1-C	primer extension	sense [a]	TAATACGACTCACTATAGG
		antisense	GTCCATGGAGGAAATCATCTTTCATCTCCAGGCTGGTGAACCTATA GTGAGTCGTATTA
A1-D	primer extension	sense [a]	TAATACGACTCACTATAGG
		antisense	TGGAGGAAATCATCTTTCATCTCCACCTATAGTGAGTCGTATTA
A1-E	primer extension	sense [a]	TAATACGACTCACTATAGG
		antisense	TCTGCAGTCCATAGGCTGGTGAACTGTGAGACCTATAGTGAGTCG TATTA
C7-A	primer extension	sense [a]	TAATACGACTCACTATAGG
		antisense	CGCCAGCTATCCTGAGGGAAACTTCGGAGGGAACCCGTTTCCCAG GACGAGCTCGCGCCTATAGTGAGTCGTATTA
C7-B	primer extension	sense [a]	TAATACGACTCACTATAGG
		antisense	ATCCTGAGGGAAACTTCGGAGGGAACCCGTTTCCCAGGACCTATA GTGAGTCGTATTA
C7-C	primer extension	sense [a]	TAATACGACTCACTATAGG
		antisense	GGGAAACTTCGGAGGGAACCCGTTTCCCTATAGTGAGTCGTATTA

a. Sequences are the same.

Table S3 Different types of sense and antisense RNA strands for the formation of RNA variants

Different types of RNA	Sense Strand (5´ → 3´)	Antisense Strand (5´ → 3´)
RNA-A	GCAGCUCGUCCUG [a]	CAGGAUAGCUGC [b]
RNA-B	GCAGCUCAUCCUG [c]	CAGGACAGCUGC [d]
RNA-C	GCAGCUGUCCUG	CAGGAUAGCUGC [b]
RNA-D	GCAGCUCGUCCUG [a]	CAGGAUCAGCUGC
RNA-E	GCAGCUCAUCCUG [c]	CAGGAUAGCUGC [b]
RNA-F	GCAGCUCGUCCUG [a]	CAGGACAGCUGC [d]
RNA-G	GCAGCUCUUCCUG	CAGGAAAGCUGC
RNA-H	GCAGCUCIUCCUG	CAGGAUAGCUGC [b]
RNA-I	GCAGCACGUCCUG	CAGGAUUGCUGC [e]
RNA-J	GCAGCAUGUCCUG	CAGGAUUGCUGC [e]
RNA-K	GCAGCUCGCUCUG	CAGAGUAGCUGC
RNA-L	GCACGUCGUCCUG	CAGGAUACGUGC

a, b, c, d, and e: Sequences are the same, respectively.

Table S4 Selected RNA sequences and their original sources

Sorted position in 96-well plate	RNA sequence [a]	Matched RNA transcript	Accession ID
A1	TCTCACAGTTCACCAGCCTGGAGA<br>TGAAAGATGATTTCCTCCATGGAC<br>TGCAGATGCTGAAATCTCTCCGCC<br>CCCCGTTCCCCCCTCCTCCTCGG<br>CGCGCGGCGGCGGAGAGCCGGC<br>T	Homo sapiens protein O-mannose kinase (POMK), transcript variant 1, mRNA	NM_032237.5
A10	TGATAGCTCTTTCTCGATTCCGTG<br>GGTGGTGGTGCATGGCCGTTCTC<br>TTCTTGTGATGTGACAGGATGATT<br>CATACCAGTCACTTTTAAAGCACT<br>GTGTAATGAGCAGAGTCCTTAGAA<br>ACATCTTAGCATC	Homo sapiens genomic DNA, chromosome 21q, section 10/105	AP001666.1
B3	GACCGGTCGTGTGTGGGTTGACTT<br>CGGTGAAGGCTCAACTACTTTACT<br>GGAAGGTTATACCTCTCATTACCC<br>ACACACGAAGGAAAGCAGGACCTT<br>CAGCCGTTCCGAAG	Homo sapiens CD44 molecule (Indian blood group) (CD44), transcript variant 6, mRNA	NM_0012025<br>55.2
B12	ACCTGGGACTAAAGACCTTCTTGA<br>TCCTCGTTCATAGGTTATAACATCT<br>GTTAAAATGTGAAAGCCACAGCTG<br>AAAAGGCTATGTGAATAGGCAAGA<br>CTAAACTGTACATTTTCTGGCTTGT<br>AGCCCTGTGTTCT	Homo sapiens DNA topoisomerase I (TOP1), RefSeqGene on chromosome 20	NG_012262.2
C7	CGGCGGCGACTCTGGACGCGAGC<br>TCGTCCTGGGAAACGGGTTCCCTC<br>CGAAGTTTCCCTCAGGATAGCTGG<br>CGCTCTCGCAGACCCGACGCACC<br>CCCGC	Homo sapiens RNA, 28S ribosomal N5 (RNA28SN5), ribosomal RNA	NR_003287.4
C9	TAGCTTATCAGACTGATGTTGATG<br>GTGGTCATTAAGCGGAGATCCGG<br>CCAGCGGAAGCCTGCCACCTCCT<br>ATGTGCGGACCACCATCAACAAGA<br>ATGCTCGCGCC	Homo sapiens ribosomal protein L28 (RPL28), transcript variant 2, mRNA	NM_000991.5
C10	TCTCTATTTTACCCTCCTACAAGCC<br>TCAGAGTACTTCGAGTCTCCCTTC<br>ACCATTTCCGACGGCATCTACGGC<br>TCAACATTTTTTGTAGCCACAG	Homo sapiens isolate 262_Sb haplogroup H1b2 mitochondrion, complete genome	MK617280.1
C12	ACACAATTGTAACACCTTCAGCAA<br>CGAAGTGGCACCGTCCGCCCAGC<br>GCCGAATCCCCGCCCCGCGGCGG<br>GGCGCGGGACATGTGGCGTACGG<br>AAGACC	Homo sapiens RNA, 28S ribosomal N5 (RNA28SN5), ribosomal RNA	NR_003287.4

a. Sequences were inserted between oligonucleotide primers for library preparation. The underlined regions are those matched with the transcriptome and genome database listed in the right column.

Table S5 Sequence variants of sorted RNAs

Original RNA	Variant	Sequence
A1	A	GGTCTCACAGTTCACCAGCCTGGAGATGAAAGATGATTTCCTCCATGGACTGCAGA
	B	GGCTGAAATCTCTCCGCCCCCCGTTCCCCCCTCCTCCTCGGCGCGCGGCGGCGGAGAGCCGGC
	C	GGTTCACCAGCCTGGAGATGAAAGATGATTTCCTCCATGGAC
	D	GGTGGAGATGAAAGATGATTTCCTCCA
	E	GGTCTCACAGTTCACCAGCCTATGGACTGCAGA
C7	A	GGCGCGAGCTCGTCCTGGGAAACGGGTTCCCTCCGAAGTTTCCCTCAGGATAGCTGGCG
	B	GGTCCTGGGAAACGGGTTCCCTCCGAAGTTTCCCTCAGGAT
	C	GGGAAACGGGTTCCCTCCGAAGTTTCCC

Table S6. Comparison of chemical shifts of berberine in an aqueous solution and bound to RNA-A.

Chemical shifts	BRB (ppm)	RNA-A/BRB (ppm)
H-1	7.42	6.62
H-4	6.88	6.07
H-5	3.15	2.37, 2.85
H-6	4.79	4.35, 4.75
H-8	9.55	9.41
H-11	7.96	7.70
H-12	7.87	7.03
H-13	8.42	7.44
$-OCH_2O-$	6.02	7.94, 6.16
9-OCH_3	4.03	4.04
10-OCH_3	4.02	3.91

Table S7. Statistics of the RNA-A/berberine structure

NMR Restraints	7A3Y
total NOE distance restraints	75
intranucleotide	26
internucleotide	41
RNA-ligand	8
Hydrogen-bond restraints	31
Glycosidic torsion angle restraints	25
Backbone torsion angle restraints	137
Deviations from the Idealized Geometry	
Bond lengths (Å)	0.01
Torsion angles (deg)	2.57
Pairwise Heavy Atom RMSD (Å)	
all heavy atoms	0.29
RNA	0.29
BRB	0.21

Table S8. K_{obs} values at 25 ˚C (× 10^6 M^{-1}) for the interaction of BRB with RNAs in the presence of different KCl concentrations [a]

RNA	KCl concentration				
	30 mM	100 mM	300 mM	600 mM	1000 mM
RNA-A	3.91 ± 0.62	2.79 ± 0.64	1.26 ± 0.09	0.53 ± 0.14	0.45 ± 0.10
RNA-B	1.14 ± 0.29	0.98 ± 0.25	0.79 ± 0.12	0.34 ± 0.04	0.22 ± 0.02

a. Values represent the average ± S.D. of triplicate experiments.

Table S9. Thermodynamic stability of RNA-A in the absence and presence of BRB or its analogue [a]

Thermodynamic parameters	without ligand	with BRB [b]	with COP [b]	with PAM [b]	with EBERB [b]
T_m (˚C)	51.8 ± 0.66	53.6 ± 0.06	54.6 ± 0.64	53.1 ± 0.62	52.8 ± 0.89
$\Delta H_{\text{(melting)}}$ (kcal mol^{-1})	-99.4 ± 2.3	-116 ± 1.0	-110 ± 3.0	-110 ± 1.6	-108 ± 1.4
$\Delta S_{\text{(melting)}}$ (cal mol^{-1} K^{-1})	-278 ± 7.8	-329 ± 3.1	-309 ± 9.6	-310 ± 5.4	-305 ± 5.1
$\Delta G°_{25\text{ (melting)}}$ (kcal mol^{-1})	-16.4 ± 0.06	-18.4 ± 0.11	-18.2 ± 0.14	-17.7 ± 0.08	-17.4 ± 0.21

a. Values represent the average ± S.D. obtained from triplicate experiments.
b. Ligand was added at 10 μM concentration.

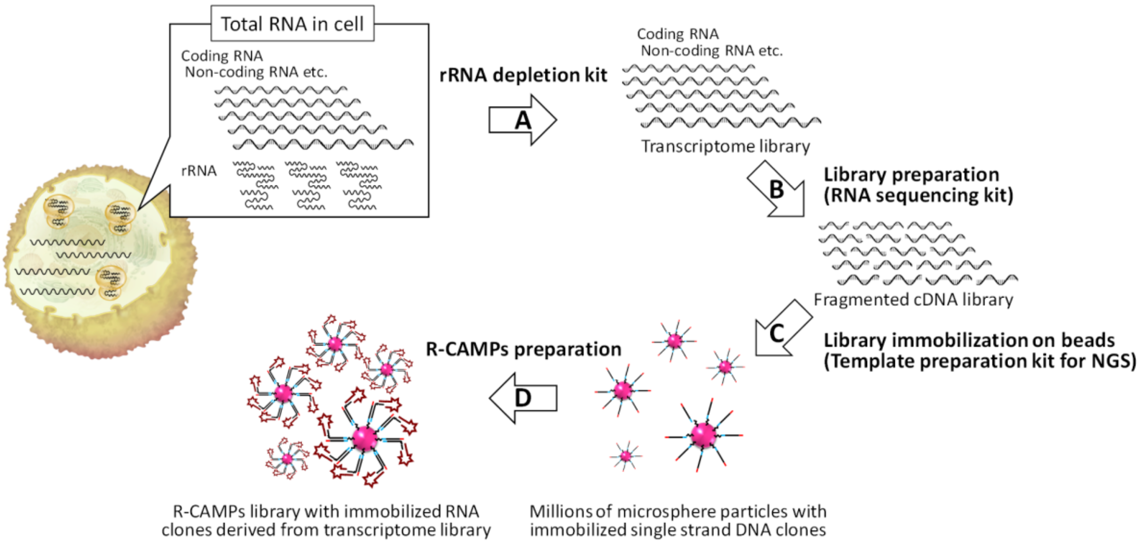

Figure S1. Process for preparing R-CAMPs immobilizing RNA clones of transcriptome library. (A) rRNA in total cellular RNA was first removed using rRNA depletion kit. (B) The transcriptome library was fragmented and transferred to a cDNA library. (C) Single-stranded DNA clones immobilized on microsphere particles. Processes from A to C are well established for transcriptome analysis using next generation sequencing. (D) RNA-capturing microsphere particles (R-CAMPs), each of which immobilizes RNA clones derived from the transcriptome library, prepared according to previously established procedure. Detailed processes for preparing R-CAMPs are described in Materials and Methods.

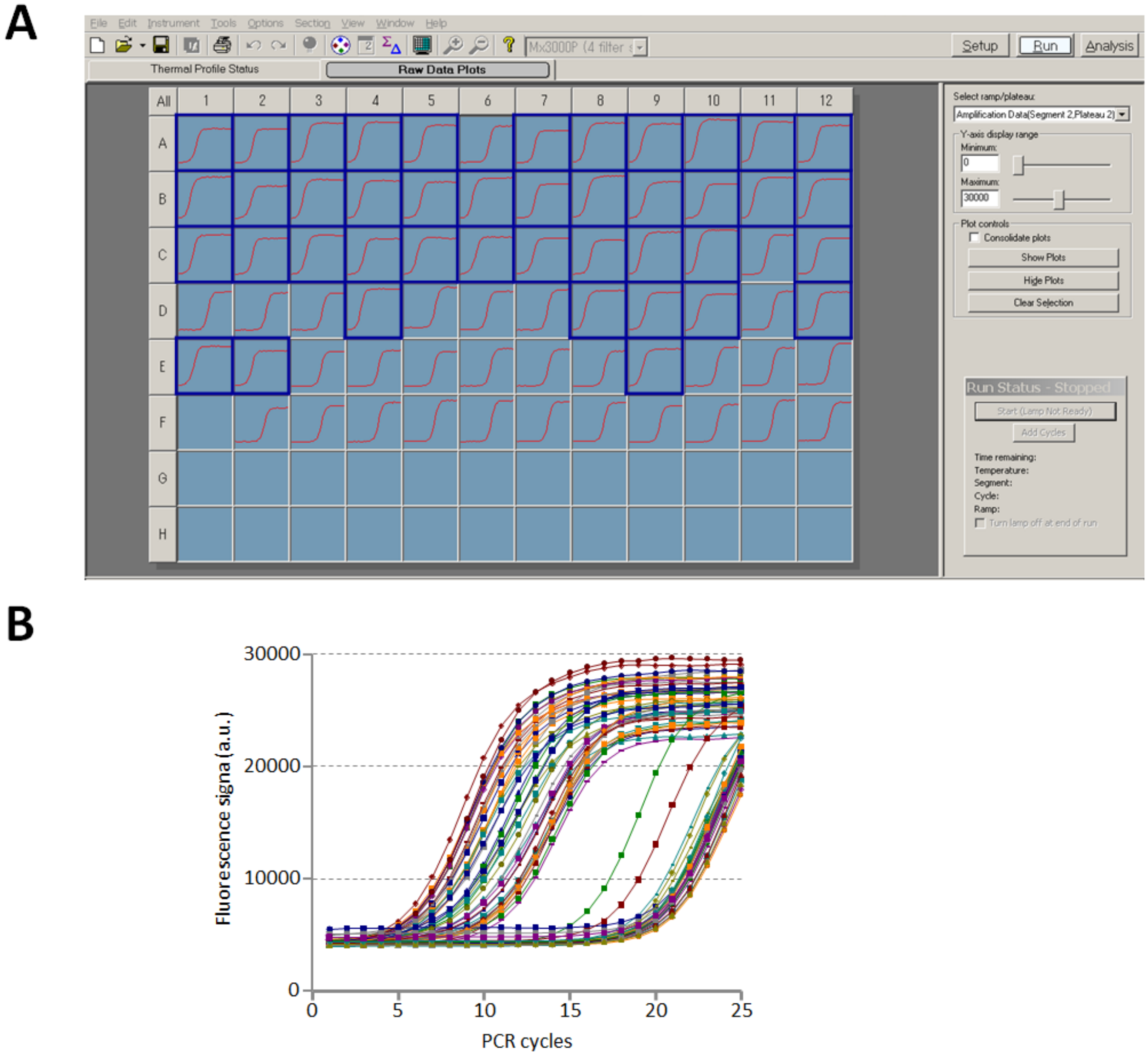

Figure S2. DNA amplification profile during quantitative real-time PCR. (A) Individual amplification profiles in 96-well format displayed on a software for real-time PCR. Letters and numbers shown at left and top, respectively, represent the well positions, at which single R-CAMP sorted into 96-well plates. R-CAMPs were sorted from A1 (upper left) to F12 (lower right). Well position at F1 (lower left) does not contain the sample. In each well position, the horizontal axis indicates the PCR cycle from 1 to 40, and the vertical axis indicates the arbitrary unit (a.u.) of the fluorescence signal (0 to 30000). Red lines denote each amplification profile. Well positions with over 10000 a.u. after 15 cycles of PCR are denoted by blue squares. (B) Fluorescence signals of 71 well positions during PCR (1–25 cycle) plotted on the PCR cycle.

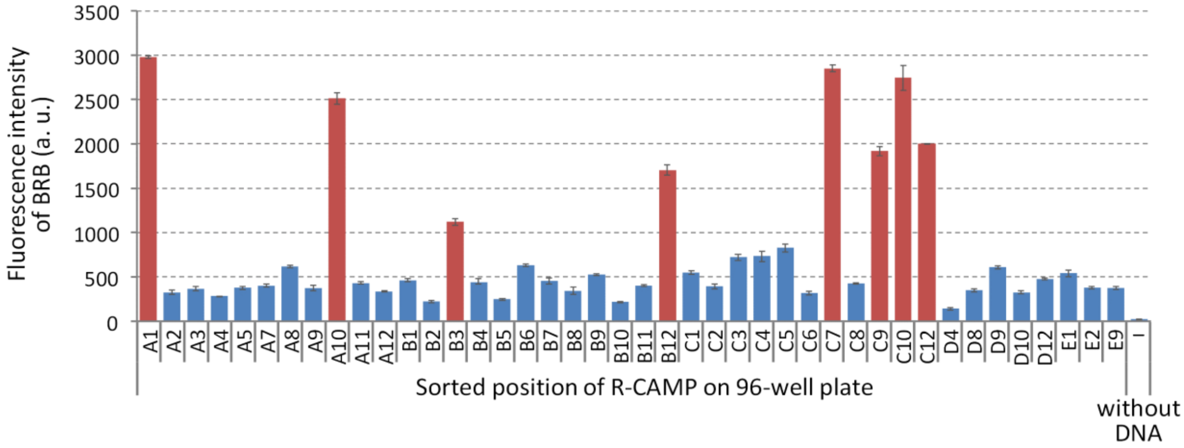

Figure S3. Fluorescence signals of BRB mixed with RNA transcripts. RNAs were transcribed from DNA templates amplified from sorted single particle, and directly diluted into a buffer containing 50 mM MES-LiOH (pH 7), 100 mM KCl, 0.5 mM $MgCl_2$, 0.1% DMSO, 0.01% Tween-20, and 1 μM BRB. The fluorescence intensities of BRB in the reaction mixtures were measured using 365 nm excitation and 550 nm emission after incubation at 25ºC for 60 min. The reaction buffer contains Tris, DTT, rNTP, T7 RNA polymerase, and DNase derived from the dilution of the transcription buffer. Letters and numbers of the RNA samples denote the well positions, at which single particles were sorted into 96-well plates. Values and errors represent the average ± S.D. of triplicate experiments. RNA transcripts derived from well positions of A1, A10, B3, B12, C7, C9, C10, and C12 (red bars) showed significantly higher fluorescence signals than other RNA samples.

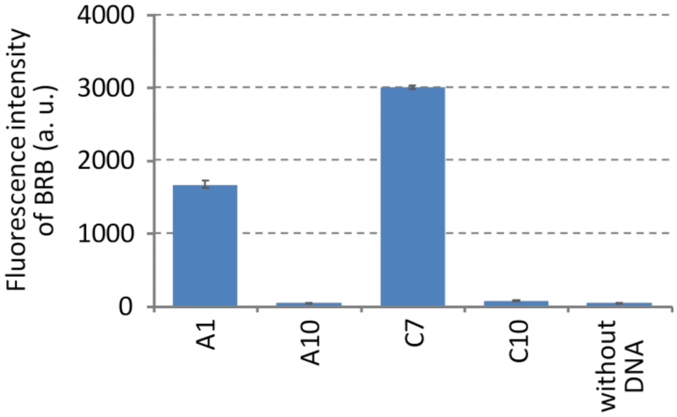

Figure S4. Fluorescence signals of BRB mixed with RNA transcripts. RNAs consisting of the sequences in Table S1 and consecutive two guanines at 5'-terminus were transcribed from DNA templates prepared by PCR amplification using oligonucleotides. RNA transcripts were directly diluted into a buffer containing 50 mM MES-LiOH (pH 7), 100 mM KCl, 0.5 mM $MgCl_2$, 0.1% DMSO, 0.01% Tween-20, and 1 μM BRB. The fluorescence intensities of BRB in the reaction mixtures were measured using 365 nm excitation and 550 nm emission after incubation at 25ºC for 60 min. The reaction buffer contains Tris, DTT, rNTP, T7 RNA polymerase, and DNase derived from the dilution of the transcription buffer. Values and errors represent the average ± S.D. of triplicate experiments.

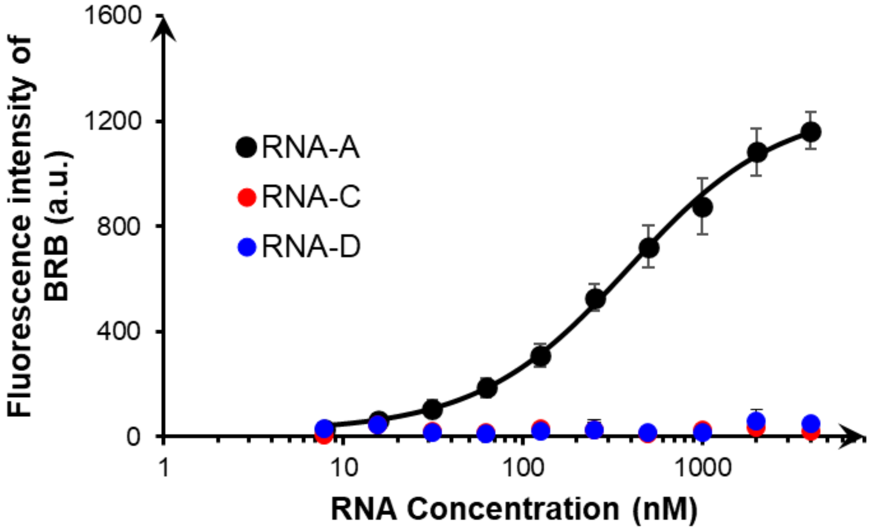

Figure S5. The fluorescence intensities of BRB mixed with RNA variants. BRB (50 nM) was mixed with various concentrations of RNA-A (black), RNA-C (red), or RNA-D (blue) in a buffer containing 50 mM MES-LiOH (pH 7), 0.5 mM $MgCl_2$, 100 mM KCl, 0.1% DMSO, and 0.01% Tween-20. The fluorescence signals of BRB were measured at 25ºC after 60-min incubation using 365 nm excitation and 550 nm emission. Values and errors represent the average ± S.D. of triplicate experiments. Plots with RNA-A are the same with those in Figure 3.

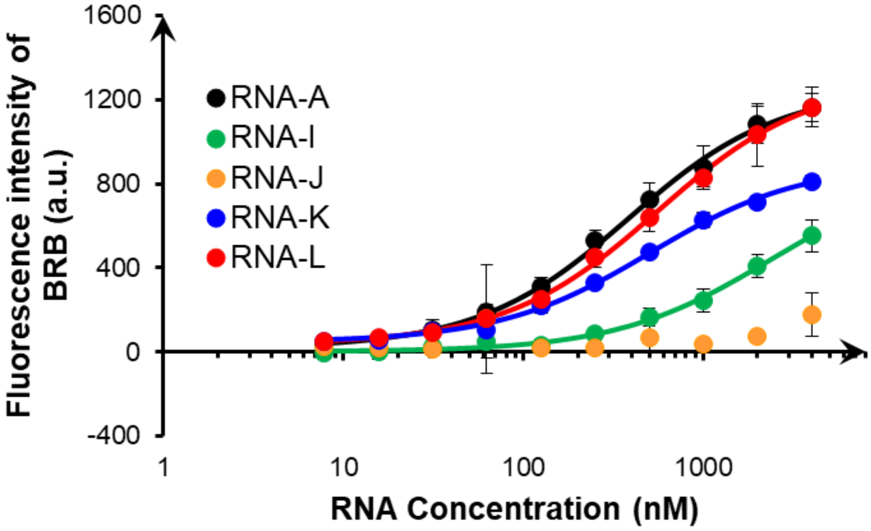

Figure S6. The fluorescence intensities of BRB mixed with RNA variants. BRB (50 nM) was mixed with various concentrations of RNA-A (black), RNA-I (green), RNA-J (orange), RNA-K (blue), and RNA-L (red) in a buffer containing 50 mM MES-LiOH (pH 7), 0.5 mM $MgCl_2$, 100 mM KCl, 0.1% DMSO, and 0.01% Tween-20. The fluorescence signals of BRB were measured at 25ºC after 60-min incubation using 365 nm excitation and 550 nm emission. Values and errors represent the average ± S.D. of triplicate experiments. Plots with RNA-A are the same with those in Figure 3.

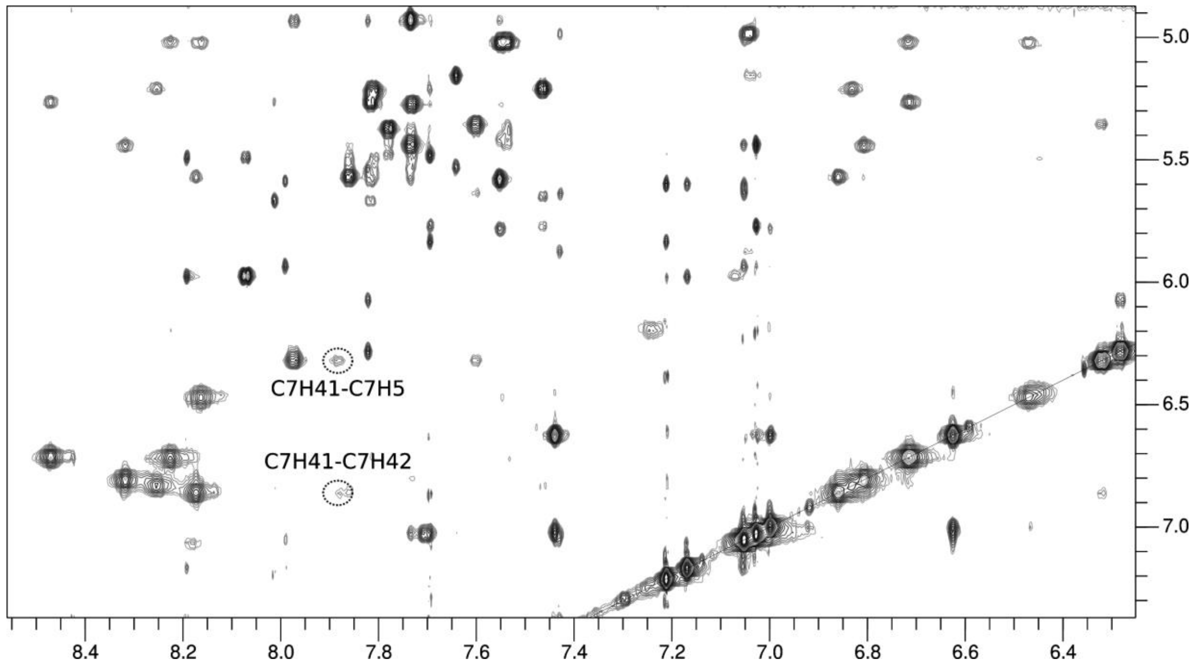

Figure S7. 2D NOESY NMR spectrum (τ_m = 250 ms) of the RNA-A/BRB complex at 25 °C. C7's hydrogen bonded amino proton (H41) was assigned via two contacts to the non-hydrogen bonded H42 and aromatic H5 protons. The two cross-peaks are assigned in the spectrum.

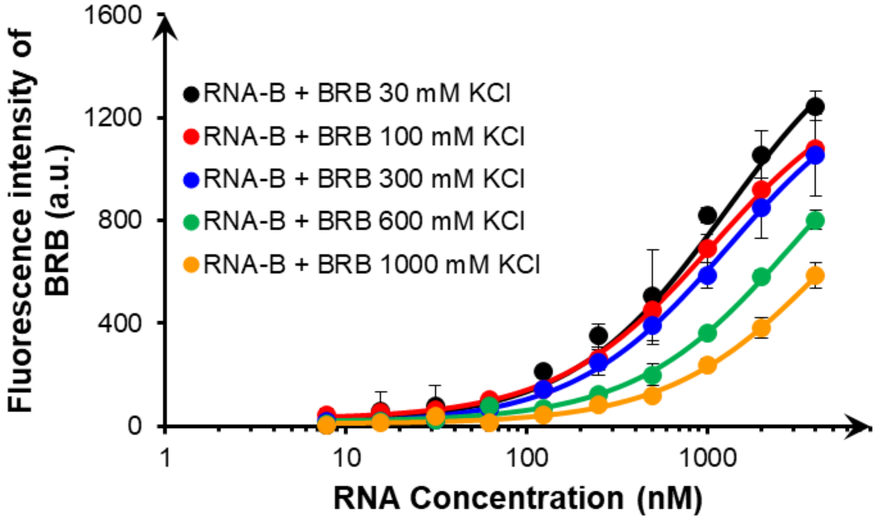

Figure S8. Effect of KCl concentration on the interaction between BRB and RNA-B. BRB (50 nM) was mixed with various concentrations of RNA-B in a buffer containing 50 mM MES-LiOH (pH 7), 0.5 mM $MgCl_2$, 0.1% DMSO, and 0.01% Tween-20 in the presence of 30 mM (black), 100 mM (red), 300 mM (blue), 600 mM (green), or 1000 mM (orange) KCl. Fluorescence signals of BRB were measured at 25ºC after 60-min incubation using 365 nm excitation and 550 nm emission. Values and errors represent the average ± S.D. of triplicate experiments.

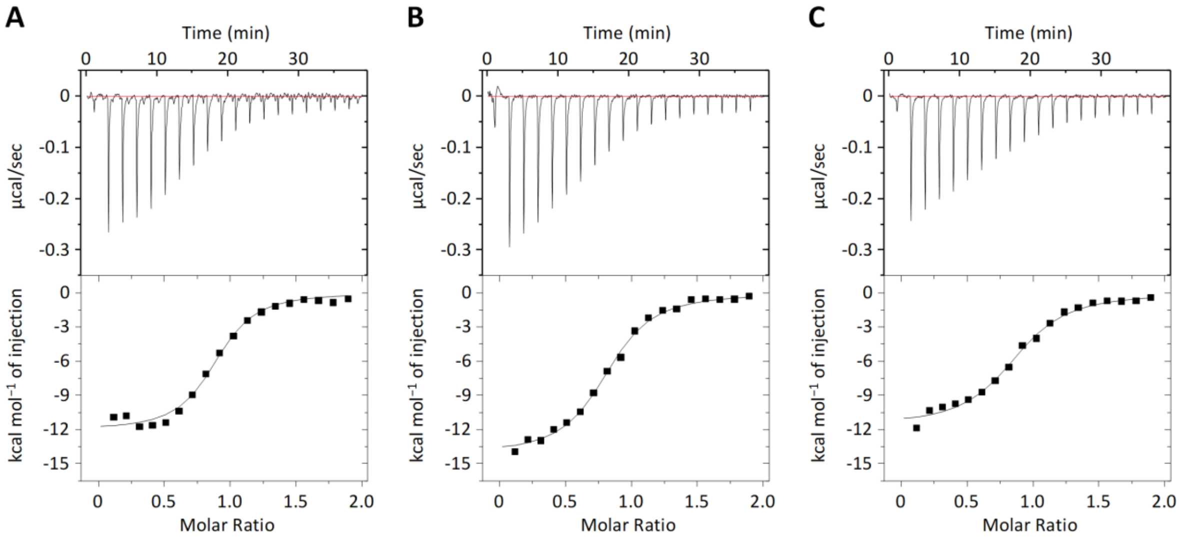

Figure S9. ITC thermograms (upper panels) and isotherms (lower panels) for the titration of RNA-A into BRB analogues. RNA-A (100 µM in the syringe) was titrated into (A) COP, (B) PAM, or (C) EBRB at 10 µM in the cell in a buffer containing 10 mM Na_2HPO_4 (pH 7), 100 mM NaCl, 1 mM EDTA, and 0.1% DMSO at 25ºC. The solid lines in lower panels represent the lines fitted to a single binding site model.
